# The Optimal Error Estimate of the Fully Discrete Locally Stabilized Finite Volume Method for the Non-Stationary Navier-Stokes Problem

**DOI:** 10.3390/e24060768

**Published:** 2022-05-30

**Authors:** Guoliang He, Yong Zhang

**Affiliations:** 1 School of Mathematical Science, University of Electronic Science and Technology of China, Chengdu 610056, China; hegl@uestc.edu.cn; 2 School of Medical Information and Engineering, Southwest Medical University, Luzhou 646099, China

**Keywords:** Navier-Stokes equations, finite volume method, fully discrete, optimal error estimate

## Abstract

This paper proves the optimal estimations of a low-order spatial-temporal fully discrete method for the non-stationary Navier-Stokes Problem. In this paper, the semi-implicit scheme based on Euler method is adopted for time discretization, while the special finite volume scheme is adopted for space discretization. Specifically, the spatial discretization adopts the traditional triangle $P_{1}-P_{0}$ trial function pair, combined with macro element form to ensure local stability. The theoretical analysis results show that under certain conditions, the full discretization proposed here has the characteristics of local stability, and we can indeed obtain the optimal theoretic and numerical order error estimation of velocity and pressure. This helps to enrich the corresponding theoretical results.

## 1. Introduction

Recently, due to the characteristics of simple implementation, the finite volume method has been widely used in many scientific research and engineering fields. It has obtained many ideal numerical simulation and calculation results, and often used to solve the complex engineering calculation problems well. Nevertheless, compared with a wide range of application scenarios, its theoretical analysis, such as stability and convergence analysis, is far behind, which inevitably shadow benefits of the finite volume method, so it needs to be studied continuously. Among them, the theoretical analysis of Navier Stokes process is one of the important field.

Finite volume method is an effective method to solve differential equations. In the past several decades, the calculation methods used to solve Navier-Stokes problems have developed rapidly. The results are richer and richer, but to our dismay, the theoretical analysis of the algorithm is still insufficient [1,2]. As we all know, based on the current advanced computing equipment, simple numerical methods are easy to distribute and suitable for large-scale computing, which makes them the hope of solving complex problems, such as incompressible Navier-Stokes equations. Among them, using simple coordinated low-order elements and local stabilization method is a good choice [3,4].

However, it is well known that low order coordinated finite volume function pairs, such as $P_{1}-P_{0}$, are unstable for numerical solution of Navier-Stokes equations. The common way to overcome this shortage is to use local stabilization technique, that is, add “macro element condition” to improve the stability of the algorithm. This kind of low order method has been widely analyzed and applied, and has been proved to be effective in practice [5,6,7,8,9]. The basic idea of this method was first proposed by Boland and Nicolaides, and has been vigorously developed since then. The recent work of Wen [10] and Li [11] paves the way for the numerical analysis of Stokes and Navier Stokes problems. In addition, He [12] and Li [13] have given the locally stable finite volume method for partial spatial discretization of Navier-Stokes problems, which has a good effect except some fully discrete results.

This paper continues to analyze the convergence results of using FVM to solve two-dimensional time-dependent Navier-Stokes equations, so as to enrich the relevant theories. Here we will still focus on $P_{1}-P_{0}$ pairs. For this purpose, let’s assume $T_{h}$ is the uniform and regular triangulation of $\bar{\Omega }$. It should be reminded that the finite element space here does not have the inf-sup condition for $\left(x_{h},m_{h}\right)$, so a similar skill in the paper [11,12,13] is required. Because these papers mainly discuss the spatial discrete case, this paper studies a approximation based on time-discretization is Euler semi-implicit and space-discretization is $P_{1}-P_{0}$ Locally stable FVM.

For brevity, this paper assumes $T_{h}$ is a regular triangulation that satisfies the general regular condition [14,15]. Let the mesh size is *h* and the time step is 0<Δt<1, the theoretical results of the optimal order convergence of the fully discrete FVM based on the low order coordinated finite element local stabilization are as follows:

For such a finite volume solution $\left(u_{h}^{n},p_{h}^{n}\right)$ obtained by the fully discrete locally stabilized FVM, we derive in this paper the following order error estimates:

(1) $$\Delta t\sum_{k=1}^{N}∥{\tilde{A}}_{h}^{\frac{1}{2}}\left(u\left(t_{k}\right)-u_{h}^{k}\right){∥}_{0}^{2}+\tau \left(t_{n}\right){∥{\tilde{A}}_{h}^{\frac{1}{2}}\left(u\left(t_{n}\right)-u_{h}^{n}\right)∥}_{0}^{2}\leq \kappa \left(h^{2}+\Delta t\right),$$

(2) $$\begin{array}{c} \Delta t\sum_{k=1}^{N}\tau \left(t_{k}\right)∥p\left(t_{k}\right)-p_{h}^{k}{∥}_{0}^{2}+\tau^{2}\left(t_{n}\right){∥p\left(t_{n}\right)-p_{h}^{n}∥}_{0}^{2}\leq \kappa \left(h^{2}+\Delta t\right), \end{array}$$

where $N=\frac{T}{\Delta t},t_{n}=n\Delta t\in \left(0,T\right],\tau \left(t\right)=min\left\{1,t\right\}$.

The rest of the paper is organized as follows. Section 2 introduces some basic concepts and function definitions related to Navier-Stokes problem and the locally stable FVM. Some basic results are prepared in Section 3. Section 4 mainly analyzes the error estimation of time semi-discretization based on Euler semi-implicit scheme. Section 5 proves the error estimations of time and spatial fully discretization. Section 6 contains some numerical results and a summary of the article is included in Section 7.

## 2. Foundation of Finite Volume Method for the Navier-Stokes Problem

This article consider the follow non-stationary Navier-Stokes equations

(3) $$\left\{\begin{array}{c} u_{t}-\nu \Delta u+\left(u\cdot \nabla \right)u+\nabla p=f,\;div\;u=0\;\forall \left(x,t\right)\in \Omega \times \left(0,T\right],\\ u\left(x,0\right)=u_{0}{\left(x\right)\;\forall x\in \Omega ;\;u\left(x,t\right)|}_{\partial \Omega }=0\;\forall t\in \left(0,T\right], \end{array}\right.$$

where Ω be a bounded domain in $R^{2}$ assumed to have a common continuous boundary ∂Ω (stronger than Lipschitz continuity) [14,16]; $u=u\left(x,t\right)=(u_{1}\left(x,t\right),u_{2}\left(x,t\right))$ are the velocity vector and p=p(x,t) is the pressure, f=f(x,t) is the body force, $u_{0}\left(x\right)$ is the initial velocity, and ν>0 is the viscosity.

For the convenience of analysis of problem (3), we introduce the following common abbreviations

$$X=H_{0}^{1}{\left(\Omega \right)}^{2},\;\;Y=L^{2}{\left(\Omega \right)}^{2},\;\;M=L_{0}^{2}\left(\Omega \right)=\left\{q\in L^{2}\left(\Omega \right);\int_{\Omega }qdx=0\right\},$$

$$H=\left\{v\in Y;\mathrm{div}\;v=0,v\cdot n{|}_{\Gamma }=0\right\}.\;\;V=\left\{v\in X;div\;v=0\right\}.$$

where *V* is the closed subset of *X* and *H* is denote the closed subset of *Y*, respectively. The spaces $L^{2}{\left(\Omega \right)}^{m},m=1,2,4$, are endowed with the common norm denoted by (·,·) and ${∥\cdot ∥}_{0}$, respectively. The norms of the Hilbert space $H_{0}^{1}\left(\Omega \right)$ and *X* are

$$\left(\left(u,v\right)\right)=\left(\nabla u,\nabla v\right),\;\;\;∥u∥={\left(\left(u,u\right)\right)}^{1/2}.$$

For more information about the above marks, we refer the reader to [14,16,17]. We also need to denote A=−Δ as the Laplace operator and $\bar{A}=-P\Delta$ as the Stokes operator, where *P* is the $L^{2}$-orthogonal projection of *Y* onto *H*.

It is known [17] that

(4) $${∥Av∥}_{0}^{2}\leq {∥v∥}_{2}^{2}\leq c{∥\bar{A}v∥}_{0}^{2}\;\forall v\in H^{2}{\left(\Omega \right)}^{2}\cap V,$$

(5) $${∥v∥}_{0}^{2}\leq \gamma_{0}{∥v∥}^{2}\;\forall v\in {X,\;\;∥v∥}^{2}\leq \gamma_{0}{∥v∥}_{2}^{2}\leq c{∥Av∥}_{0}^{2}\;\forall v\in D\left(A\right),$$

where $D\left(A\right)=H^{2}{\left(\Omega \right)}^{2}\cap X$, $\gamma_{0}$ is positive constant depending only on Ω. *C*, like the quantities $C_{i},i=1,2,\cdots$, appear subsequently, is a positive constant depending on Ω.

This paper uses following kind of continuous bilinear forms a(·,·) and d(·,·) on X×X and X×M, respectively,

a(u,v)=ν((u,v))∀u,v∈X,

d(v,q)=−(v,∇q)=(q,divv)∀v∈X,q∈M.

and a trilinear form on X×X×X

$$\begin{array}{cc} b(u,v,w) & =\frac{1}{2}\left(\left(u\cdot \nabla \right)v,w\right)-\frac{1}{2}\left(\left(u\cdot \nabla \right)w,v\right)\;\;\forall u,v,w\in X. \end{array}$$

With the above notations, the general variational formulation of problem (3) is: get $\left(u,p\right)\in L^{\infty }\left(0,T;H\right)\cap L^{2}\left(0,T;X\right)\times L^{2}\left(0,T;M\right)$ satisfy:

(6) $$\left\{\begin{array}{c} \left(u_{t},v\right)+a\left(u,v\right)-d\left(v,p\right)+d\left(u,q\right)+b\left(u,u,v\right)=\left(f,v\right),\;\;t\in \left(0,T\right],\\ u\left(0\right)=u_{0}. \end{array}\right.$$

for all (v,q)∈(X,M).

In order to get the fully dispersed error estimates, we need the following smoothness.

**Theorem** **1.** *Assume some continuity of $u_{0}$ and f(x) are valid [14,17]. Then problem (6) admits a unique solution (u,p) satisfying the following estimates:*

(7) $$\begin{array}{c} {∥u\left(t\right)∥}^{2}+\int_{0}^{t}\left(∥u_{t}{∥}_{0}^{2}+{∥Au∥}_{0}^{2}+{∥p∥}_{1}^{2}\right)ds\leq \kappa ,\\ \tau \left(t\right)\left({∥Au\left(t\right)∥}_{0}^{2}+{∥p\left(t\right)∥}_{1}^{2}+{∥u_{t}\left(t\right)∥}_{0}^{2}\right)+\int_{0}^{t}\tau \left(s\right){∥u_{t}∥}^{2}ds\leq \kappa ,\\ \tau^{2}\left(t\right)∥u_{t}{\left(t\right)∥}^{2}+\int_{0}^{t}\tau^{2}\left(s\right)(∥u_{tt}{∥}_{0}^{2}+∥Au_{t}{∥}_{0}^{2}+∥p_{t}{∥}_{1}^{2})ds\leq \kappa , \end{array}$$

*for all t∈[0,T], where κ is a positive constant.*

Now, we consider the fully discrete locally stabilized FVM for two-dimensional time-dependent incompressible Navier-Stokes Equation (3). For convenience, let $\tau_{h}=\tau_{h}\left(\Omega \right)$ a partitioning of $\bar{\Omega }$ into triangles satisfied the regular in the usual sense (see [5,14]). $\left(X_{h},M_{h}\right)$ are the corresponding finite element subspace of (X,M). $\Gamma_{h}$ is the set of all interelement boundaries.

**Figure 1:**
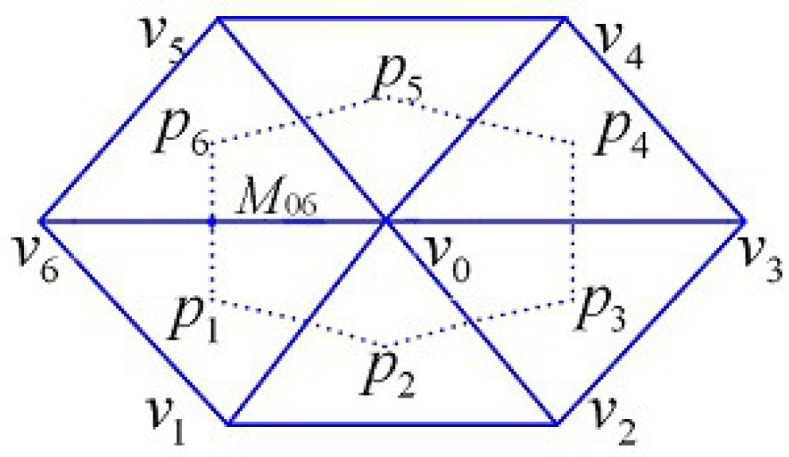
The finite volume partition of geometric region.

In order to define the finite volume method for Equation (3), We need introduce a popular configuration of dual partition $T_{h}^{*}$ for $T_{h}$: the interior point $p_{i}$ is chosen to the barycenter of element $K_{i}\in T_{h}$, and the midpoint $m_{ij}$ on side of $\bar{v_{i}v_{j}}$. See Figure 1. This type of dual partition is locally regular if $T_{h}$ is locally regular.

Corresponding to Hilbert space V,H, we define the following finite element velocity subspaces

$$X_{h}=\left\{v\in X:\;v_{i}{|}_{K}\in P_{1}\left(K\right),\;\;i=1,2\;\;\;\forall K\in \tau_{h}\right\},$$

and the pressure subspace

$$M_{h}=\left\{q\in M:\;\;\;q{|}_{K}\in P_{0}\left(K\right)\;\;\;\forall K\in \tau_{h}\right\}.$$

The finite volume dual of velocity is $X_{h}^{*}$

$$X_{h}^{*}=\left\{v\in L^{2}{\left(\Omega \right)}^{2}:\;v_{i}{|}_{K^{*}}\in P_{0}\left(K^{*}\right),\;\;i=1,2\;\;\;\forall K^{*}\in T_{h}^{*}\right\}.$$

Let interpolation operator $I_{h}^{*}$:$X_{h}\to X_{h}^{*}$.

$$I_{h}^{*}u_{h}=\sum_{x_{i}\in N_{h}}u_{h}\left(x_{i}\right)\chi_{i}\left(x\right).$$

The $L^{2}$ dimensional reduction $P_{h}:X\to X_{h}$ and $J_{h}:M\to M_{h}$ are defined as follows:

(8) $$\left(P_{h}v,v_{h}\right)=\left(v,v_{h}\right)\;\;\forall v\in Y,\;v_{h}\in X_{h},\;\left(J_{h}q,q_{h}\right)=\left(q,q_{h}\right)\;\;\forall q\in M,\;q_{h}\in M_{h}.$$

The finite volume forms of velocity $\tilde{a}\left(\cdot ,\cdot \right)$ on $X_{h}\times X_{h}$ is,

$$\tilde{a}\left(u_{h},I_{h}^{*}v_{h}\right)=\nu \left(\left(u_{h},I_{h}^{*}v_{h}\right)\right)=-\nu \sum_{K_{i}^{*}\in T_{h}^{*}}\int_{\partial K_{i}^{*}}v_{h}\left(x_{i}\right)\frac{\partial u_{h}}{\partial n}ds\;\;\;\forall u_{h},v_{h}\in X_{h},$$

where n is the unit outnormal vector. The finite volume form $\tilde{d}\left(\cdot ,\cdot \right)$ of pressure on $X_{h}\times M_{h}$ is defined as

$$\tilde{d}\left(I_{h}^{*}v_{h},p_{h}\right)=-\left(I_{h}^{*}v_{h},\nabla p_{h}\right)=\sum_{K_{i}^{*}\in T_{h}^{*}}\int_{\partial K_{i}^{*}}p_{h}\;v_{h}\left(x_{i}\right)\cdot nds\;\;\;\forall u_{h}\in X_{h}\;,\;p_{h}\in M_{h},$$

To facilitate the analysis, we need the following two trilinear forms.

$$\begin{array}{cc} \tilde{b}\left(u_{h},v_{h},I_{h}^{*}w_{h}\right)=\left(\left(u_{h}\cdot \nabla \right)v_{h},I_{h}^{*}w_{h}\right)\; & +\frac{1}{2}\left(\left(\mathrm{div}u_{h}\right)v_{h},I_{h}^{*}w_{h}\right),\\ \bar{b}\left(u_{h},v_{h},w_{h}-I_{h}^{*}w_{h}\right)=\left(\left(u_{h}\cdot \nabla \right)v_{h},I_{h}^{*}w_{h}-I_{h}^{*}w_{h}\right)\; & +\frac{1}{2}\left(\left(\mathrm{div}u_{h}\right)v_{h},w_{h}-I_{h}^{*}w_{h}\right)\\ \; & \;\forall u_{h},v_{h},w_{h}\in X_{h}, \end{array}$$

The last time difference part is

$$\left(u_{ht},I_{h}^{*}v_{h}\right)=\sum_{K_{i}^{*}\in T_{h}^{*}}\int_{K_{i}^{*}}v_{h}\left(x_{i}\right)\;u_{ht}dx\;\;\;\forall v_{h}\in X_{h}.$$

The finite volume form of the right side is

$$\left(f,I_{h}^{*}v_{h}\right)=\sum_{K_{i}^{*}\in T_{h}^{*}}\int_{K_{i}^{*}}v_{h}\left(x_{i}\right)\;fdx\;\;\;\forall v_{h}\in X_{h}.$$

For the convenience of reading, we introduce the following generalized form

$$\tilde{B}\left(\left(u_{h},p_{h}\right);\left(I_{h}^{*}v_{h},q_{h}\right)\right)=\tilde{a}\left(u_{h},I_{h}^{*}v_{h}\right)-\tilde{d}\left(I_{h}^{*}v_{h},p_{h}\right)+d\left(u_{h},q_{h}\right).$$

**Figure 2:**
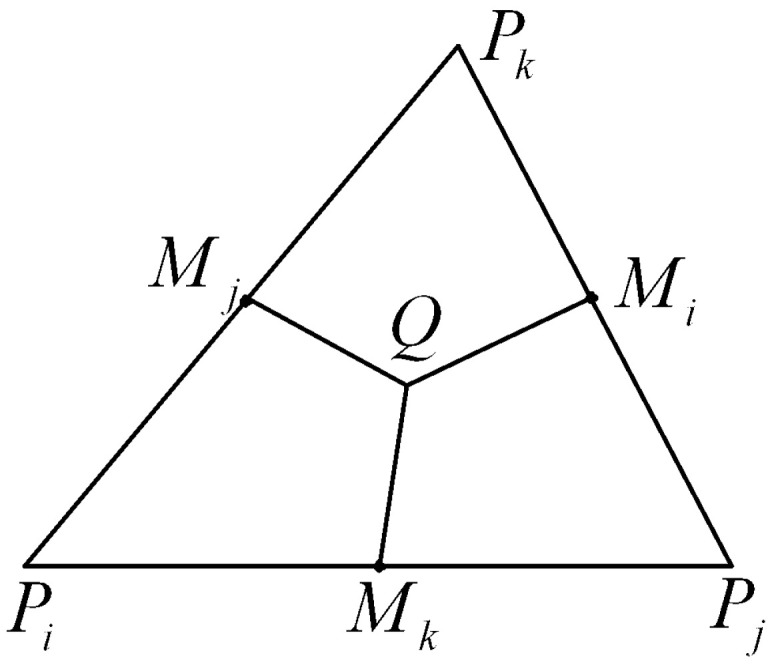
The area of partition of triangular.

In this paper, the norms are defined as following:

$$\begin{array}{cc} ∥u_{h}{∥}_{0,h,K} & =\begin{array}{c} {\left[\frac{S_{Q}}{3}\left(u_{P_{i}}^{2}+u_{P_{j}}^{2}+u_{P_{k}}^{2}\right)\right]}^{1/2}, \end{array}\\ ∥{\tilde{A}}_{h}^{1/2}u_{h}{∥}_{0,h,K} & =\begin{array}{c} {\left\{\left[{\left(\frac{\partial u_{h}\left(Q\right)}{\partial x}\right)}^{2}+{\left(\frac{\partial u_{h}\left(Q\right)}{\partial y}\right)}^{2}\right]S_{Q}\right\}}^{1/2}, \end{array} \end{array}$$

$$\begin{array}{cc} & ∥u_{h}{∥}_{0,h}={\left(\sum_{K\in T_{h}}{∥u_{h}∥}_{0,h,K}^{2}\right)}^{1/2},\\ & ∥{\tilde{A}}_{h}^{1/2}u_{h}{∥}_{0}={\left(\sum_{K\in T_{h}}{∥{\tilde{A}}_{h}^{1/2}u_{h}∥}_{0,h,K}^{2}\right)}^{1/2},\\ & ∥u_{h}{∥}_{1,h}={\left(∥u_{h}{∥}_{0,h}^{2}+{∥{\tilde{A}}_{h}^{1/2}u_{h}∥}_{0}^{2}\right)}^{1/2}, \end{array}$$

where $S_{Q}$ is the area of $▵P_{i}P_{j}P_{k}$ (see Figure 2).

To describe the locally stabilized formulation of the non-stationary Navier-Stokes problem, we use the classic not overlap *macroelement partitioning* $\Lambda_{h}$ [18]. For every macroelement K in $\Lambda_{h}$, the set of interelement(small finite element) edges is denoted by $\Gamma_{K}$, and the length of an edge $e\in \Gamma_{K}$ is denoted by $h_{e}$.

With the above definitions, a locally stabilized formulation of the non-stationary Navier-Stokes problem (3) can be stated as follows.

**Definition** **1.** *Locally stabilized finite volume formulation for non-stationary Navier-Stokes: Find $\left(u_{h},p_{h}\right)\in \left(X_{h},M_{h}\right),$ such that for all $\left(v,q\right)\in \left(X_{h},M_{h}\right)$*

(9) $$\left\{\begin{array}{c} \left(u_{ht},I_{h}^{*}v\right)+{\tilde{B}}_{h}\left(\left(u_{h},p_{h}\right);\left(I_{h}^{*}v,q\right)\right)+\tilde{b}\left(u_{h},u_{h},I_{h}^{*}w_{h}\right)=\left(f,I_{h}^{*}v\right),\\ u_{h}\left(0\right)=u_{0h}. \end{array}\right.$$

*where*

$${\tilde{B}}_{h}\left(\left(u_{h},p_{h}\right);\left(I_{h}^{*}v,q\right)\right)=\tilde{B}\left(\left(u_{h},p_{h}\right);\left(I_{h}^{*}v,q\right)\right)+\beta \;C_{h}\left(p_{h},q\right)\;\;\forall \;\left(u_{h},p_{h}\right),\;\left(v,q\right)\in \left(X_{h},M_{h}\right),$$

$$C_{h}\left(p,q\right)=\sum_{K\in \Lambda_{h}}\sum_{e\in \Gamma_{K}}h_{e}\int_{e}{\left[p\right]}_{e}{\left[q\right]}_{e}ds,$$

*for all p,q in the algebraic sum $H^{1}\left(\Omega \right)+M_{h}$, and ${\left[\cdot \right]}_{e}$ is the jump operator across $e\in \Gamma_{K}$ and β>0 is the local stabilization parameter.*

In order get the regularity of the above definitions, we need the following stability results [5,8,9,12].

**Theorem** **2.** *For any two neighboring macroelements $K_{1}$ and $K_{2}$ with $\int_{K_{1}\cap K_{2}}ds\neq 0$, if there exists $v\in X_{h}$ such that*

(10) $$suppv\subset K_{1}\cup K_{2}\;\mathrm{and}\;\int_{K_{1}\cap K_{2}}v\cdot nds\neq 0.$$

*Then*

(11) $$\begin{array}{cc} |{\tilde{B}}_{h}\left(\left(u,p\right);\left(I_{h}^{*}v,q\right)\right)| & \leq c(∥u∥+{∥p∥}_{0})(∥v∥+{∥q∥}_{0})\;\;\forall \left(u,p\right),\left(v,q\right)\in \left(X,M\right),\\ \alpha (∥{\tilde{A}}_{h}^{\frac{1}{2}}u_{h}{∥}_{0}+∥p_{h}{∥}_{0}) & \leq \underset{\left(v_{h},q_{h}\right)\in \left(X_{h},M_{h}\right)}{\sup}\frac{{\tilde{B}}_{h}\left(\left(u_{h},p_{h}\right);\left(I_{h}^{*}v_{h},q_{h}\right)\right)}{∥{\tilde{A}}_{h}^{\frac{1}{2}}v_{h}{∥}_{0}+{∥q_{h}∥}_{0}} \end{array}$$

*for all $\left(u_{h},p_{h}\right)\in \left(X_{h},M_{h}\right)$, and*

(12) $$C_{h}\left(p,q_{h}\right)=0,\;C_{h}\left(p_{h},q\right)=0,\;C_{h}\left(p,q\right)=0\;\;\forall p,\;q\in H^{1}\left(\Omega \right),\;p_{h},\;q_{h}\in M_{h},$$

*where $\beta \geq \beta_{0}>0$ and α>0 are two constant.*

## 3. Technical Preliminaries

The main task of this section is to prepare many basic estimates which will help the error analyses for the finite volume solution $\left(u_{h},p_{h}\right)$.

Since the bilinear forms $\left(\left(u_{h},v_{h}\right)\right)$ and $\left(\left(u_{h},I_{h}^{*}v_{h}\right)\right)$ are coercive on $X_{h}\times X_{h}$, they generate invertible operators $A_{h}:X_{h}\to X_{h}$ and ${\tilde{A}}_{h}:X_{h}\to X_{h}$ respectively through the condition:

$$\begin{array}{c} \left(A_{h}u_{h},v_{h}\right)=\left(A_{h}^{1/2}u_{h},A_{h}^{1/2}v_{h}\right)=\left(\left(u_{h},v_{h}\right)\right)\;\;\forall u_{h},v_{h}\in X_{h},\\ \left({\tilde{A}}_{h}u_{h},I_{h}^{*}v_{h}\right)=\left(\left(u_{h},I_{h}^{*}v_{h}\right)\right)\;\;\forall u_{h},v_{h}\in X_{h}. \end{array}$$

Moreover, we also need the discrete gradient operators:

$$\left(v_{h},\nabla_{h}q_{h}\right)=-d\left(v_{h},q_{h}\right)\;\;\forall \left(v_{h},q_{h}\right)\in \left(X_{h},M_{h}\right),$$

Firstly, we have the following classical properties (see [8,19])

(13) $$\begin{array}{cc} ∥v_{h}∥\leq ch^{-1}∥v_{h}{∥}_{0},\;\;{∥v_{h}∥}_{\infty } & \leq {c|\ln h|}^{1/2}∥v_{h}∥\;\;\forall v_{h}\in X_{h},\\ ∥P_{h}v∥ & \leq c∥v∥\;\;\forall v\in X.\\ ∥v-I_{h}{v∥}_{0}+h∥v-I_{h}v∥ & \leq ch^{2}{∥v∥}_{2}\;\forall v\in D\left(A\right),\\ ∥v-I_{h}^{*}{v∥}_{0} & \leq ch∥v∥\;\forall v\in X, \end{array}$$

where ${∥v∥}_{\infty }={∥v∥}_{L^{\infty }}={\mathrm{ess}.\sup}_{x\in \Omega }{∥v\left(x\right)∥}_{0}$. For the $P_{1}-P_{0}$ triangular element, It follows from (4), (5) and (13) that [20,21]

(14) $$\begin{array}{c} ∥v_{h}{∥}_{0}\leq \gamma_{0}∥v_{h}∥,\;\;∥v_{h}∥\leq \gamma_{0}∥A_{h}v_{h}{∥}_{0},\;\;∥A_{h}v_{h}{∥}_{0}\leq ch^{-1}∥v_{h}∥\;\;\forall v_{h}\in X_{h},\\ \;∥v_{h}{∥}_{0,h}\leq \gamma_{0}^{\prime }∥{\tilde{A}}_{h}^{\frac{1}{2}}v_{h}{∥}_{0},\;∥{\tilde{A}}_{h}^{\frac{1}{2}}v_{h}{∥}_{0}\leq \gamma_{0}^{\prime }{∥{\tilde{A}}_{h}v_{h}∥}_{0}\;\;\forall v_{h}\in X_{h},\\ ∥{\tilde{A}}_{h}^{\frac{1}{2}}v_{h}{∥}_{0}\leq ch^{-1}∥v_{h}{∥}_{0,h},\;\;∥{\tilde{A}}_{h}v_{h}{∥}_{0}\leq ch^{-1}{∥{\tilde{A}}_{h}^{\frac{1}{2}}v_{h}∥}_{0}\;\;\forall v_{h}\in X_{h},\\ \;c_{1}∥v_{h}{∥}_{0}\leq ∥v_{h}{∥}_{0,h}\leq c_{1}^{\prime }∥v_{h}{∥}_{0},\;\;c_{1}∥A_{h}v_{h}{∥}_{0}\leq ∥{\tilde{A}}_{h}v_{h}{∥}_{0}\leq c_{1}^{\prime }{∥A_{h}v_{h}∥}_{0}\;\;\forall v_{h}\in X_{h}. \end{array}$$

As for the trilinear forms $\tilde{b}$ and $\bar{b}$,we can deduce the following results.

**Lemma** **1.** *If $u_{h},v_{h},w_{h}\in X_{h}$, we have*

(15) $$\begin{array}{c} \begin{array}{cc} |\tilde{b}\left(u_{h},v_{h},I_{h}^{*}w_{h}\right)| & +\;|\bar{b}\left(u_{h},v_{h},w_{h}-I_{h}^{*}w_{h}\right)|\leq c_{2}∥{\tilde{A}}_{h}^{\frac{1}{2}}u_{h}{∥}_{0}∥{\tilde{A}}_{h}^{\frac{1}{2}}v_{h}{∥}_{0}{∥{\tilde{A}}_{h}^{\frac{1}{2}}w_{h}∥}_{0},\\ |\tilde{b}\left(u_{h},v_{h},I_{h}^{*}w_{h}\right)| & \leq c_{2}∥u_{h}{∥}_{0}∥{\tilde{A}}_{h}^{\frac{1}{2}}v_{h}{∥}_{0}∥{\tilde{A}}_{h}^{\frac{1}{2}}w_{h}{∥}_{0}^{1/2}{∥{\tilde{A}}_{h}w_{h}∥}_{0}^{\frac{1}{2}},\\ |\tilde{b}\left(u_{h},v_{h},I_{h}^{*}w_{h}\right)| & \leq c_{2}∥{\tilde{A}}_{h}^{\frac{1}{2}}u_{h}{∥}_{0}^{1/2}∥{\tilde{A}}_{h}u_{h}{∥}_{0}^{\frac{1}{2}}∥{\tilde{A}}_{h}^{\frac{1}{2}}v_{h}{∥}_{0}{∥w_{h}∥}_{0}.\\ |\tilde{b}\left(u_{h},v_{h},I_{h}^{*}w_{h}\right)| & \leq c_{2}{\left|\ln h\right|}^{\frac{1}{2}}∥{\tilde{A}}_{h}^{\frac{1}{2}}u_{h}{∥}_{0}∥{\tilde{A}}_{h}^{\frac{1}{2}}v_{h}{∥}_{0}{∥w_{h}∥}_{0}, \end{array} \end{array}$$

**Proof.**  Since the following discrete analogue of the Sobolev inequality holds [17]

(16) $$∥\phi_{h}{∥}_{L^{4}}\leq c∥\phi_{h}{∥}_{0}^{1/2}{∥A_{h}^{1/2}\phi_{h}∥}_{0}^{1/2}\;\forall \phi_{h}\in X_{h}.$$

If $u_{h},v_{h},w_{h}\in X_{h},$, we have

$$\left|\tilde{b}\left(u_{h},v_{h},I_{h}^{*}w_{h}\right)\right|\leq c∥u_{h}{∥}_{L^{4}}∥A_{h}^{1/2}v_{h}{∥}_{0}{∥w_{h}∥}_{L^{4}},$$

combining the above formula with (14) and (16), we can get the first formula in (15).

To prove the others in (15), we need the follow discrete results [17,22], namely for any h>0,

(17) $$\begin{array}{cc} ∥\phi_{h}{∥}_{L^{\infty }} & \leq c_{3}{\left|\ln h\right|}^{1/2}∥v_{h}∥,\\ ∥\phi_{h}{∥}_{L^{6}} & \leq c_{3}∥\phi_{h}∥\;\;\forall \phi_{h}\in X_{h},\\ ∥\phi_{h}{∥}_{L^{\infty }}+{∥\nabla \phi_{h}∥}_{L^{3}} & \leq c_{3}∥\phi_{h}{∥}^{1/2}{∥A_{h}\phi_{h}∥}_{0}^{1/2}\;\;\forall \phi_{h}\in X_{h}. \end{array}$$

For any $u_{h},v_{h},w_{h}\in X_{h}$, we have [9]

$$\begin{array}{ccc} |\tilde{b}\left(u_{h},v_{h},I_{h}^{*}w_{h}\right)| & \leq & c_{4}∥u_{h}{∥}_{L^{\infty }}∥{\tilde{A}}_{h}^{\frac{1}{2}}v_{h}{∥}_{0}∥w_{h}{∥}_{0}+c_{4}∥\nabla u_{h}{∥}_{L^{3}}∥v_{h}{∥}_{L^{6}}{∥w_{h}∥}_{0},\\ |\tilde{b}\left(v_{h},u_{h},I_{h}^{*}w_{h}\right)| & \leq & c_{4}∥v_{h}{∥}_{L^{6}}∥\nabla u_{h}{∥}_{L^{3}}∥w_{h}{∥}_{0}+c_{4}∥{\tilde{A}}_{h}^{\frac{1}{2}}v_{h}{∥}_{0}∥u_{h}{∥}_{L^{\infty }}{∥w_{h}∥}_{0},\\ |\tilde{b}\left(u_{h},v_{h},I_{h}^{*}w_{h}\right)| & \leq & c_{4}∥u_{h}{∥}_{0}∥{\tilde{A}}_{h}^{\frac{1}{2}}v_{h}∥∥w_{h}{∥}_{L^{\infty }}+c_{4}∥u_{h}{∥}_{0}∥\nabla v_{h}{∥}_{L^{3}}{∥w_{h}∥}_{L^{6}}, \end{array}$$

which together with (14), (17) imply (15).  □

Similar to the results in [12], we also need to define the projection operator $\left({\tilde{R}}_{h},{\tilde{Q}}_{h}\right):\left(X,Y\right)\to \left(X_{h},M_{h}\right)$ as

$$\begin{array}{c} {\tilde{B}}_{h}\left(\left({\tilde{R}}_{h}\left(u,p\right),{\tilde{Q}}_{h}\left(u,p\right)\right);\left(I_{h}^{*}v_{h},q_{h}\right)\right)={\tilde{B}}_{h}\left(\left(u,p\right);\left(I_{h}^{*}v_{h},q_{h}\right)\right)\\ \;\forall \left(u,p\right)\in \left(X,M\right),\;\left(v_{h},q_{h}\right)\in \left(X_{h},M_{h}\right). \end{array}$$

Due to Theorem 2, we know that $\left({\tilde{R}}_{h},{\tilde{Q}}_{h}\right)$ is well defined and have the properties [12]:

(18) $$∥{\tilde{A}}_{h}^{\frac{1}{2}}\left({\tilde{R}}_{h}\left(u,p\right)-u\right){∥}_{0}+∥{\tilde{Q}}_{h}{\left(u,p\right)-p∥}_{0}\leq {ch(∥u∥}_{2}+{∥p∥}_{1}),$$

for all $\left(u,p\right)\in (H^{2}{\left(\Omega \right)}^{2}\cap X,\;H^{1}\left(\Omega \right)\cap M)$.

Beside, we need the specific result in He et al. [12].

**Theorem** **3.** *Under the assumptions of Theorems 1 and 2, $\left(u_{h},p_{h}\right)$ satisfies*

(19) $$\int_{0}^{t}∥{\tilde{A}}_{h}^{\frac{1}{2}}\left(u-u_{h}\right){∥}_{0}^{2}ds+\tau \left(t\right)∥{\tilde{A}}_{h}^{\frac{1}{2}}\left(u\left(t\right)-u_{h}\left(t\right)\right){∥}_{0}^{2}+\tau^{2}\left(t\right){∥p\left(t\right)-p_{h}\left(t\right)∥}_{0}^{2}\leq \kappa h^{2},$$

*for all t∈[0,T].*

Since our error analysis for the time discretization depends heavily on these regularity estimates, we then provide some smoothness estimates of $\left(u_{h},p_{h}\right)$. The main idea is similar to the work in He et al. [9,23].

**Theorem** **4.** *Under the assumptions of Theorems 3, the finite volume solution $\left(u_{h},p_{h}\right)$ satisfies*

(20) $$\begin{array}{c} \begin{array}{cc} ∥{\tilde{A}}_{h}^{\frac{1}{2}}u_{h}{\left(t\right)∥}_{0}^{2} & +\int_{0}^{t}(∥{\tilde{A}}_{h}^{-1}\left(u_{htt}+\nabla_{h}p_{ht}\right){∥}_{0}^{2}+∥u_{ht}{∥}_{0}^{2}+∥{\tilde{A}}_{h}u_{h}{∥}_{0}^{2})ds\leq \kappa ,\\ \tau^{2}\left(t\right){∥{\tilde{A}}_{h}^{\frac{1}{2}}u_{ht}\left(t\right)∥}^{2} & +\int_{0}^{\tau }\tau^{2}\left(s\right)(∥u_{htt}+\nabla_{h}p_{ht}{∥}_{0}^{2}+∥{\tilde{A}}_{h}u_{ht}{∥}_{0}^{2})ds\leq \kappa ,\\ \tau^{2}\left(t\right){∥{\tilde{A}}_{h}^{\frac{1}{2}}u_{ht}\left(t\right)∥}^{2} & +\int_{0}^{\tau }\tau^{2}\left(s\right)(∥u_{htt}+\nabla_{h}p_{ht}{∥}_{0}^{2}+∥{\tilde{A}}_{h}u_{ht}{∥}_{0}^{2})ds\leq \kappa , \end{array} \end{array}$$

*for all t∈[0,T].*

**Proof.**  Note from (13) and (14) we can deduce the following estimates:

$$\begin{array}{cc} ∥{\tilde{A}}_{h}^{\frac{1}{2}}u_{h}{\left(t\right)∥}_{0} & \leq ∥{\tilde{A}}_{h}^{\frac{1}{2}}\left(u_{h}\left(t\right)-A_{h}^{1/2}P_{h}u\left(t\right)\right){∥}_{0}+c∥u\left(t\right)∥\\ \; & \leq ch^{-1}∥u_{h}{\left(t\right)-u\left(t\right)∥}_{0}+c∥u\left(t\right)∥,\\ ∥{\tilde{A}}_{h}u_{h}{∥}_{0} & =\underset{v_{h}\in X_{h}}{\sup}\frac{\left|({\tilde{A}}_{h}u_{h},I_{h}^{*}v_{h})\right|}{∥I_{h}^{*}v_{h}{∥}_{0}}\leq (ch^{-1}∥{\tilde{A}}_{h}^{\frac{1}{2}}\left(u-u_{h}\right){∥}_{0}+{∥Au∥}_{0}). \end{array}$$

and from Theorems 1 and 3, we have

$$\begin{array}{cc} ∥{\tilde{A}}_{h}^{\frac{1}{2}}u_{h}{\left(t\right)∥}_{0}^{2} & +\int_{0}^{t}{∥{\tilde{A}}_{h}u_{h}∥}_{0}^{2}ds\leq c\left({∥u\left(t\right)∥}^{2}+\int_{0}^{t}{∥Au∥}_{0}^{2}ds\right)\\ \; & +ch^{-2}\left(∥u\left(t\right)-u_{h}{\left(t\right)∥}_{0,h}^{2}+\int_{0}^{t}{∥{\tilde{A}}_{h}^{\frac{1}{2}}\left(u-u_{h}\right)∥}_{0}^{2}ds\right)\leq \kappa ,\\ \;\;\tau \left(t\right)∥{\tilde{A}}_{h}u_{h}{\left(t\right)∥}_{0}^{2} & \leq c\tau \left(t\right)(h^{-2}∥{\tilde{A}}_{h}^{\frac{1}{2}}\left(u\left(t\right)-u_{h}\left(t\right)\right){∥}_{0}^{2}+∥Au\left(t\right){∥}_{0}^{2})\leq \kappa , \end{array}$$

for all t∈[0,T]. Then using the similar method in [9], we can get these estimates (20).  □

Finally, in order to get the upper bounders of velocity and pressure in the time related case, we state the classical Gronwall lemma used in [24].

**Lemma** **2.** *Let $C_{0}$ and $a_{k},b_{k},c_{k},d_{k}$, for integers k≥0, be nonnegative numbers such that*

(21) $$a_{n}+\Delta t\sum_{k=0}^{n}b_{k}\leq \Delta t\sum_{k=0}^{n-1}d_{k}a_{k}+\Delta t\sum_{k=0}^{n-1}c_{k}+C_{0}\;\;\forall n\geq 1.$$

*Then,*

(22) $$a_{n}+\Delta t\sum_{k=0}^{n}b_{k}\leq \exp\left(\Delta t\sum_{k=0}^{n-1}d_{k}\right)\left(\Delta t\sum_{k=0}^{n-1}c_{k}+C_{0}\right)\;\;\forall n\geq 1.$$

The following is dual Gronwall lemma.

**Lemma** **3.** *Given integer m>0 and let C and $a_{k},b_{k},c_{k},d_{k}$, for integers 0≤k≤m, be nonnegative numbers such that*

(23) $$a_{n}+\Delta t\sum_{k=n+1}^{m}b_{k}\leq \Delta t\sum_{k=n+1}^{m}d_{k}a_{k}+\Delta t\sum_{k=n+1}^{m}c_{k}+C,\;\;0\leq n\leq m.$$

*Then,*

(24) $$\;\;a_{n}+\Delta t\sum_{k=n+1}^{m}b_{k}\leq \exp\left(\Delta t\sum_{k=n+1}^{m}d_{k}\right)\left(\Delta t\sum_{k=n+1}^{m}c_{k}+C\right),\;\;0\leq n\leq m.$$

## 4. Error Estimates for Semi-Discrete for Time Depended Navier-Stokes Equations

In this section we consider the time discretization of the locally stabilized and get some useful estimates. Let *T* time to stop calculation and *N* the time corresponding step. So we have

$$\Delta t=\frac{T}{N},\;\;\;\;t_{n}=n\Delta t,\;\;n=0,1,\cdots ,N.$$

For the first part, we need to analysis the errors of finite element Original case. It’s well known that the common Euler semi-implicit scheme applied to the spatially discrete problem (9) can be described as:

(25) $$\begin{array}{c} \left(d_{t}u_{h}^{n},I_{h}^{*}v_{h}\right)+{\tilde{B}}_{h}\left(\left(u_{h}^{n},p_{h}^{n}\right);\left(I_{h}^{*}v_{h},q_{h}\right)\right)+\tilde{b}\left(u_{h}^{n-1},u_{h}^{n},I_{h}^{*}v_{h}\right)=\left(f^{n},I_{h}^{*}v_{h}\right), \end{array}$$

for all $\left(v_{h},q_{h}\right)\in \left(X_{h},M_{h}\right)$, where $u_{h}^{0}=u_{0h}$ is starting value and

$$d_{t}u_{h}^{n}=\frac{1}{\Delta t}\left(u_{h}^{n}-u_{h}^{n-1}\right),\;\;f^{n}=\frac{1}{\Delta t}\int_{t_{n-1}}^{t_{n}}f\left(t\right)dt,$$

$${\bar{p}}_{h}\left(t_{n}\right)=\frac{1}{\Delta t}\int_{t_{n-1}}^{t_{n}}p_{h}\left(t\right)dt.$$

To deduce the discretization error $\left(e_{h}^{n},\mu_{h}^{n}\right)=\left(u_{h}\left(t_{n}\right)-u_{h}^{n},{\bar{p}}_{h}\left(t_{n}\right)-p_{h}^{n}\right)$, we integrate and differentiate (9), respectively, to get

(26) $$\begin{array}{c} \begin{array}{cc} \frac{1}{\Delta t}\left(u_{h}\left(t_{n}\right)-u_{h}\left(t_{n-1}\right),I_{h}^{*}v_{h}\right) & +\frac{1}{\Delta t}\int_{t_{n-1}}^{t_{n}}{\tilde{B}}_{h}\left(\left(u_{h}\left(t\right),p_{h}\left(t\right)\right);\left(I_{h}^{*}v_{h},q_{h}\right)\right))dt\\ & +\frac{1}{\Delta t}\int_{t_{n-1}}^{t_{n}}\tilde{b}\left(u_{h}\left(t\right),u_{h}\left(t\right),I_{h}^{*}v_{h}\right)dt=\left(f^{n},I_{h}^{*}v_{h}\right), \end{array} \end{array}$$

(27) $$\begin{array}{c} \begin{array}{cc} \left(u_{htt}+\nabla_{h}p_{ht},I_{h}^{*}v_{h}\right)+\tilde{a}\left(u_{ht},I_{h}^{*}v_{h}\right) & -\tilde{d}\left(I_{h}^{*}v_{h},u_{ht}\right)+d\left(u_{ht},q_{h}\right){+}_{h}\left(p_{ht},q_{h}\right)\\ & +\tilde{b}\left(u_{ht},u_{h},I_{h}^{*}v_{h}\right)+\tilde{b}\left(u_{h},u_{ht},I_{h}^{*}v_{h}\right)=\left(f_{t},I_{h}^{*}v_{h}\right), \end{array} \end{array}$$

for all $\left(v_{h},q_{h}\right)\in \left(X_{h},M_{h}\right)$.

Subtracting (25) from (26) and using (27) and the relation:

$$\begin{array}{c} \phi \left(t_{n}\right)-\frac{1}{\Delta t}\int_{t_{n-1}}^{t_{n}}\phi \left(t\right)dt=\frac{1}{\Delta t}\int_{t_{n-1}}^{t_{n}}\left(t-t_{n-1}\right)\phi_{t}\left(t\right)dt \end{array}$$

for all $\phi \in H^{1}\left(t_{n-1},t_{n};F\right)$ for some Hilbert space *F*, we have

(28) $$\begin{array}{c} \begin{array}{cc} \left(d_{t}e_{h}^{n},v_{h}\right) & +{\tilde{B}}_{h}\left(\left(e_{h}^{n},\mu_{h}^{n}\right);\left(I_{h}^{*}v_{h},q_{h}\right)\right)+\tilde{b}\left(e_{h}^{n-1},u_{h}\left(t_{n}\right),I_{h}^{*}v_{h}\right)\\ & +\tilde{b}\left(u_{h}^{n-1},e_{h}^{n},I_{h}^{*}v_{h}\right)=\left(E_{n},I_{h}^{*}v_{h}\right), \end{array} \end{array}$$

for all $\left(v_{h},q_{h}\right)\in \left(X_{h},M_{h}\right)$, where $\left(e_{h}^{0},\mu_{h}^{0}\right)=\left(0,0\right)$ and

(29) $$\begin{array}{c} \begin{array}{cc} (E_{n},I_{h}^{*}v_{h})= & -\frac{1}{\Delta t}\int_{t_{n-1}}^{t_{n}}\left(t-t_{n-1}\right)\left(u_{htt}+\nabla_{h}p_{ht},I_{h}^{*}v_{h}\right)dt\\ & +\tilde{b}\left(\int_{t_{n-1}}^{t_{n}}u_{ht}dt,u_{h}\left(t_{n}\right),I_{h}^{*}v_{h}\right). \end{array} \end{array}$$

**Lemma** **4.** *Under the assumptions of Theorem 3 the error $E_{n}$ satisfies the following bounds:*

(30) $$\begin{array}{c} \begin{array}{cc} \Delta t\sum_{n=1}^{m}{∥{\tilde{A}}_{h}^{-1}E_{n}∥}_{0}^{2} & \leq \kappa \Delta t^{2},\;\;1\leq m\leq N,\\ \Delta t\sum_{n=1}^{m}\tau^{i}\left(t_{n}\right){∥{\tilde{A}}_{h}^{-1/2}E_{n}∥}_{0}^{2} & \leq \kappa \Delta t^{i+1},\;\;1\leq m\leq N,\;\;i=0,1,\\ \Delta t\sum_{n=1}^{m}\tau^{i}\left(t_{n}\right){∥E_{n}∥}_{0,h}^{2} & \leq \kappa \Delta t^{i},\;\;1\leq m\leq N,\;\;i=0,1,2. \end{array} \end{array}$$

**Proof.**  By using (14), (15) and (29), we derive

(31) $$\begin{array}{c} \begin{array}{cc} ∥{\tilde{A}}_{h}^{-1}E_{n}{∥}_{0} & \leq \frac{1}{\Delta t}\int_{t_{n-1}}^{t_{n}}\left(t-t_{n-1}\right)∥{\tilde{A}}_{h}^{-1}\left(u_{htt}+\nabla_{h}p_{ht}\right){∥}_{0}dt+c∥{\tilde{A}}_{h}^{\frac{1}{2}}u_{h}\left(t_{n}\right){∥}_{0}\int_{t_{n-1}}^{t_{n}}{∥u_{ht}∥}_{0}dt\\ & \leq c\Delta t^{1/2}{\left(\int_{t_{n-1}}^{t_{n}}\left(∥{\tilde{A}}_{h}^{-1}\left(u_{htt}+\nabla_{h}p_{ht}\right){∥}_{0}^{2}+∥{\tilde{A}}_{h}^{\frac{1}{2}}u_{h}\left(t_{n}\right){∥}_{0}^{2}{∥u_{ht}∥}_{0}^{2}\right)dt\right)}^{1/2}. \end{array} \end{array}$$

Applying Theorem 4 in (39), we obtain

(32) $$\begin{array}{cc} \Delta t∥{\tilde{A}}_{h}^{-1}E_{n}{∥}_{0}^{2} & \leq c\Delta t^{2}\int_{t_{n-1}}^{t_{n}}\left(∥{\tilde{A}}_{h}^{-1}\left(u_{htt}+\nabla_{h}p_{ht}\right){∥}_{0}^{2}+{∥u_{ht}∥}_{0}^{2}\right)dt. \end{array}$$

Utilizing Theorem 4, if we sum (32) from n=1 to n=m, we can derive the first inequality in (30) directly.

Next, we deduce from (19) and (29) that

(33) $$\begin{array}{c} \begin{array}{cc} ∥{\tilde{A}}_{h}^{-1/2}E_{n}{∥}_{0} & \leq \frac{1}{\Delta t}\int_{t_{n-1}}^{t_{n}}\left(t-t_{n-1}\right){∥{\tilde{A}}_{h}^{-1/2}\left(u_{htt}+\nabla_{h}p_{ht}\right)∥}_{0}dt\\ & +c\int_{t_{n-1}}^{t_{n}}∥{\tilde{A}}_{h}u_{h}\left(t_{n}\right){∥}_{0}{∥u_{htt}∥}_{0}dt\\ & \leq c(\int_{t_{n-1}}^{t_{n}}\left(\left(t-t_{n-1}\right){∥{\tilde{A}}_{h}^{-1/2}\left(u_{htt}+\nabla_{h}p_{ht}\right)∥}_{0}^{2}\right.\\ & +\Delta t\left.∥{\tilde{A}}_{h}u_{h}\left(t_{n}\right){∥}_{0}^{2}{∥u_{ht}∥}_{0}^{2}\right)dt{)}^{1/2}. \end{array} \end{array}$$

Because

(34) $$\begin{array}{c} \tau \left(t_{n}\right)\leq \tau \left(t\right)+\Delta t,\;\;\Delta t\leq \tau \left(t_{n}\right),\;\;t-t_{n-1}\leq \tau \left(t\right),\;\;\forall t\in \left[t_{n-1},t_{n}\right], \end{array}$$

We can deduce from (33) and Theorem 4 to get

(35) $$\begin{array}{c} \tau^{i}\left(t_{n}\right)∥{\tilde{A}}_{h}^{-1/2}E_{n}{∥}_{0}^{2}\Delta t\leq c\Delta t^{1+i}\int_{t_{n-1}}^{t_{n}}(\tau \left(t\right)∥{\tilde{A}}_{h}^{-1/2}\left(u_{htt}+\nabla_{h}p_{ht}\right){∥}_{0}^{2}+∥u_{ht}{∥}_{0}^{2})dt, \end{array}$$

for i=0,1. Summing (35) from n=1 to n=m, we derive the second inequality in (30).

As for the last one, Deriving from (14) and (29), we have

(36) $$\begin{array}{c} \begin{array}{cc} ∥E_{n}{∥}_{0,h} & \leq \frac{1}{\Delta t}\int_{t_{n-1}}^{t_{n}}\left(t-t_{n-1}\right){∥u_{htt}+\nabla_{h}p_{ht}∥}_{0}dt\\ & +c∥{\tilde{A}}_{h}u_{h}\left(t_{n}\right){∥}_{0}{∥{\tilde{A}}_{h}^{\frac{1}{2}}\left(u_{h}\left(t_{n}\right)-u_{h}\left(t_{n-1}\right)\right)∥}_{0}. \end{array} \end{array}$$

Hence, Formula (36) and Theorem 4 imply that

(37) $$\begin{array}{c} \begin{array}{cc} \tau^{i}\left(t_{n}\right){∥E_{n}∥}_{0,h}^{2}\Delta t & \leq c\tau^{i}\left(t_{n}\right)\int_{t_{n-1}}^{t_{n}}{\left(t-t_{n-1}\right)}^{2}{∥u_{htt}+\nabla_{h}p_{ht}∥}_{0}^{2}dt\\ & +c\tau^{i}\left(t_{n}\right)∥{\tilde{A}}_{h}u_{h}\left(t_{n}\right){∥}_{0}^{2}{∥{\tilde{A}}_{h}^{\frac{1}{2}}\left(u_{h}\left(t_{n}\right)-u_{h}\left(t_{n-1}\right)\right)∥}_{0}^{2}\Delta t. \end{array} \end{array}$$

Similarly, summing (37) from n=1 to n=m and using Theorem 4 deduces

$$\begin{array}{c} \begin{array}{cc} \Delta t\sum_{n=1}^{m}\tau^{i}\left(t_{n}\right){∥E_{n}∥}_{0,h}^{2} & \leq c\sum_{n=1}^{m}\tau^{i}\left(t_{n}\right)\int_{t_{n-1}}^{t_{n}}{\left(t-t_{n-1}\right)}^{2}{∥u_{htt}+\nabla_{h}p_{ht}∥}_{0}^{2}dt\\ \; & +c\tau^{i}\left(t_{1}\right)∥{\tilde{A}}_{h}u_{h}\left(t_{1}\right){∥}_{0}^{2}{∥{\tilde{A}}_{h}^{\frac{1}{2}}\left(u_{h}\left(t_{1}\right)-u_{h}\left(t_{0}\right)\right)∥}_{0}^{2}\Delta t\\ & +c\Delta t^{2}\sum_{n=2}^{m}\tau^{i}\left(t_{n}\right)∥{\tilde{A}}_{h}u_{h}\left(t_{n}\right){∥}_{0}^{2}\int_{t_{n-1}}^{t_{n}}{∥{\tilde{A}}_{h}^{\frac{1}{2}}u_{ht}∥}_{0}^{2}dt\\ & \leq c\Delta t^{i}\sum_{n=1}^{m}\int_{t_{n-1}}^{t_{n}}\tau^{2}\left(t\right){∥u_{htt}+\nabla_{h}p_{ht}∥}_{0}^{2}dt\\ & +c∥{\tilde{A}}_{h}^{\frac{1}{2}}\left(u_{h}\left(t_{1}\right)-u_{h}\left(t_{0}\right)\right){∥}_{0}^{2}\Delta t^{i}+c\Delta t^{i}\sum_{n=2}^{m}\int_{t_{n-1}}^{t_{n}}\tau \left(t\right){∥{\tilde{A}}_{h}^{\frac{1}{2}}u_{ht}∥}_{0}^{2}dt\\ & \leq \kappa \Delta t^{i}, \end{array} \end{array}$$

for *i* = 0,1,2, which yields the third one in (30).  □

Now, let’s discuss the second part: the error of time discrete duality argument corresponding to (25). Firstly, the dual problem corresponding to (25) usually describes as: find $\left(\Phi_{h}^{n-1},\Psi_{h}^{n-1}\right)\in \left(X_{h},M_{h}\right)$ such that, for all $\left(v_{h},q_{h}\right)\in \left(X_{h},M_{h}\right),$

(38) $$\begin{array}{cc} \left(v_{h},I_{h}^{*}\left(d_{t}\Phi_{h}^{n}\right)\right) & -{\tilde{B}}_{h}\left(\left(v_{h},q_{h}\right);\left(I_{h}^{*}\Phi_{h}^{n-1},\Psi_{h}^{n-1}\right)\right)\\ \; & -\tilde{b}\left(v_{h},u_{h}\left(t_{n}\right),I_{h}^{*}\Phi_{h}^{n-1}\right)-\tilde{b}\left(u_{h}\left(t_{n-1}\right),v_{h},I_{h}^{*}\Phi_{h}^{n-1}\right)=\left(v_{h},I_{h}^{*}z^{n}\right), \end{array}$$

where $\Phi_{h}^{m}=0$.

For the dual part, we also need the existence, uniqueness and regularity of problem (38), so we introduce the following results:

$$\begin{array}{cc} ∥P_{h}B\left(u_{h}\left(t_{n}\right),\cdot \right){∥}_{*} & =\underset{v_{h}\in X_{h}}{\sup}\frac{∥P_{h}B\left(u_{h}\left(t_{n}\right),v_{h}\right){∥}_{0}}{∥{\tilde{A}}_{h}^{\frac{1}{2}}v_{h}{∥}_{0}},\\ ∥P_{h}B\left(\cdot ,u_{h}\left(t_{n}\right)\right){∥}_{*} & =\underset{v_{h}\in X_{h}}{\sup}\frac{∥P_{h}B\left(v_{h},u_{h}\left(t_{n}\right)\right){∥}_{0}}{∥{\tilde{A}}_{h}^{\frac{1}{2}}v_{h}{∥}_{0}}. \end{array}$$

With the similar method in He [9], we have the following two lemmas.

**Lemma** **5.** *Assume that the assumptions of Theorem 3 is valid and Δt satisfies*

(39) $$\frac{8}{\nu }c_{2}^{2}\left|\ln h\right|\max_{t\in [0,T]}{∥{\tilde{A}}_{h}^{\frac{1}{2}}u_{h}\left(t\right)∥}_{0}^{2}\Delta t\leq 1.$$

*Then,*

(40) $$\Delta t\sum_{n=1}^{N}\left(∥P_{h}B\left(u_{h}\left(t_{n-1}\right),\cdot \right){∥}_{*}^{2}+∥P_{h}B{(\cdot ,u_{h}\left(t_{n}\right)∥}_{*}^{2}\right)\leq \kappa .$$

**Lemma** **6.** *Assume that the assumptions of Theorem 3 are valid and that Δt satisfies (39). Then, problem (38) admits a unique solution $\left(\Phi_{h}^{n-1},\Psi_{h}^{n-1}\right)\in \left(X_{h},M_{h}\right)$ for a given $\Phi_{h}^{n}$. Furthermore, the solution sequence ${\left\{\Phi_{h}^{n},\Psi_{h}^{n}\right\}}_{n=0}^{m-1}$ of problem (38) satisfies the following bound*

(41) $$\underset{0\leq r\leq m}{\sup}∥{\tilde{A}}_{h}^{\frac{1}{2}}\Phi_{h}^{r}{∥}_{0}^{2}+\Delta t\sum_{n=1}^{m-1}∥d_{t}\Phi_{h}^{n}{∥}_{0}^{2}+\Delta t\sum_{n=0}^{m-1}∥{\tilde{A}}_{h}\Phi_{h}^{n}{∥}_{0}^{2}\leq \kappa \Delta t\sum_{n=1}^{m}{∥z^{n}∥}_{0}^{2}.$$

## 5. Error Analysis for Time and Spacial Discrete

In this section we proof the upper bounds for the error $\left(e_{h}^{n},\mu_{h}^{n}\right)=\left(u_{h}\left(t_{n}\right)-u_{h}^{n},{\bar{p}}_{h}\left(t_{n}\right)-p_{h}^{n}\right)$ in $L_{2}$ and $H_{1}$ norms, and deduce the last optimal order estimates. Firstly, we have

**Lemma** **7.** *Assume that the assumptions of Theorem 3 are valid and Δt satisfies (39). Then, the error $(e_{h}^{n},\mu_{h}^{n}),1\leq n\leq N,$ satisfies the following bound:*

(42) $$∥e_{h}^{m}{∥}_{0,h}^{2}+\Delta t\sum_{n=1}^{m}\left(∥{\tilde{A}}_{h}^{\frac{1}{2}}e_{h}^{n}{∥}_{0}^{2}+\beta C_{h}\left(\mu_{h}^{n},\mu_{h}^{n}\right)\right)\leq \kappa \Delta t,\;\;1\leq m\leq N.$$

**Proof.**  Setting $v_{h}=e_{h}^{n},\;\;q_{h}=\mu_{h}^{n}$ in (28) and using (14) we obtain

(43) $$\begin{array}{cc} \frac{c_{1}}{2\Delta t}(∥e_{h}^{n}{∥}_{0}^{2} & +∥e_{h}^{n}-e_{h}^{n-1}{∥}_{0}^{2}-∥e_{h}^{n-1}{∥}_{0}^{2})+\nu ∥{\tilde{A}}_{h}^{\frac{1}{2}}e_{h}^{n}{∥}_{0}^{2}+\beta C_{h}\left(\mu_{h}^{n},\mu_{h}^{n}\right)\\ \; & +\tilde{b}\left(e_{h}^{n-1},u\left(t_{n}\right),I_{h}^{*}e_{h}^{n}\right)+\tilde{b}\left(u\left(t_{n}\right),e_{h}^{n},I_{h}^{*}e_{h}^{n}\right)\\ \; & =\left(E_{n},e_{h}^{n}\right)\leq \frac{\nu }{4}∥{\tilde{A}}_{h}^{\frac{1}{2}}e_{h}^{n}{∥}_{0}^{2}+\nu^{-1}{∥A_{h}^{-1/2}E_{n}∥}_{0}^{2}. \end{array}$$

From (14) and (15), we deduce that

$$\begin{array}{cc} |\tilde{b}\left(e_{h}^{n-1},u_{h}\left(t_{n}\right),I_{h}^{*}e_{h}^{n}\right)|\; & \leq c∥e_{h}^{n-1}{∥}_{0}∥P_{h}B\left(\cdot ,u_{h}\left(t_{n}\right)\right){∥}_{*}{∥{\tilde{A}}_{h}^{\frac{1}{2}}e_{h}^{n}∥}_{0}\\ \; & \leq \frac{\nu }{8}∥{\tilde{A}}_{h}^{\frac{1}{2}}e_{h}^{n}{∥}_{0}^{2}+\kappa ∥P_{h}B\left(\cdot ,u_{h}\left(t_{n}\right)\right){∥}_{*}^{2}{∥e_{h}^{n-1}∥}_{0}^{2},\\ |\tilde{b}\left(u\left(t_{n}\right),e_{h}^{n},I_{h}^{*}e_{h}^{n}\right)|\; & \leq c∥e_{h}^{n}{∥}_{0}∥P_{h}B\left(\cdot ,u_{h}\left(t_{n}\right)\right){∥}_{*}{∥{\tilde{A}}_{h}^{\frac{1}{2}}e_{h}^{n}∥}_{0}\\ \; & \leq \frac{\nu }{8}∥{\tilde{A}}_{h}^{\frac{1}{2}}e_{h}^{n}{∥}_{0}^{2}+\kappa ∥P_{h}B\left(\cdot ,u_{h}\left(t_{n}\right)\right){∥}_{*}^{2}{∥e_{h}^{n}∥}_{0}^{2}. \end{array}$$

Combining (43) with the above estimate yields

(44) $$\begin{array}{cc} ∥e_{h}^{n}{∥}_{0}^{2} & -∥e_{h}^{n-1}{∥}_{0}^{2}+(\nu ∥{\tilde{A}}_{h}^{\frac{1}{2}}e_{h}^{n}{∥}_{0}^{2}+\beta C_{h}\left(\mu_{h}^{n},\mu_{h}^{n}\right))\Delta t\\ \; & \leq \kappa \left(∥P_{h}B\left(\cdot ,u_{h}\left(t_{n}\right)\right){∥}_{*}^{2}(∥e_{h}^{n-1}{∥}_{0,h}^{2}+∥e_{h}^{n}{∥}_{0,h}^{2})+∥{\tilde{A}}_{h}^{-1/2}E_{n}{∥}_{0}^{2}\right)\Delta t. \end{array}$$

Summing (44) from n=1 to n=m, we obtain

(45) $$\begin{array}{cc} ∥e^{m}{∥}_{0,h}^{2} & +\Delta t\sum_{n=1}^{m}(\nu ∥{\tilde{A}}_{h}^{\frac{1}{2}}e_{h}^{n}{∥}_{0}^{2}+\beta C_{h}\left(\mu_{h}^{n},\mu_{h}^{n}\right))\\ \; & \leq \kappa \Delta t\left(\sum_{n=1}^{m}∥P_{h}B\left(\cdot ,u_{h}\left(t_{n+1}\right)\right){∥}_{*}^{2}(∥e_{h}^{n-1}{∥}_{0,h}^{2}+∥e_{h}^{n}{∥}_{0,h}^{2})+\sum_{n=1}^{m}{∥{\tilde{A}}_{h}^{-1/2}E_{n}∥}_{0}^{2}\right). \end{array}$$

Applying Lemmas 3 and 6 in (45) and by (14) yields (43).  □

**Lemma** **8.** *Under the assumptions of Lemma 7, we have*

(46) $$∥{\tilde{A}}_{h}^{\frac{1}{2}}e_{h}^{m}{∥}^{2}+\Delta t\sum_{n=1}^{m}{∥d_{t}e_{h}^{n}∥}_{0,h}^{2}\leq \kappa ,\;\;1\leq m\leq N.$$

**Proof.**  We derive from (28) that

(47) $$\begin{array}{cc} \left(d_{t}e_{h}^{n},I_{h}^{*}v_{h}\right) & +\tilde{a}\left(e_{h}^{n},I_{h}^{*}v_{h}\right)-\tilde{d}\left(I_{h}^{*}v_{h},\mu_{h}^{n}\right)+d\left(d_{t}e_{h}^{n},q_{h}\right)+\beta C_{h}\left(d_{t}\mu_{h}^{n},q_{h}\right)\\ \; & +\tilde{b}\left(e_{h}^{n-1},u_{h}\left(t_{n}\right),I_{h}^{*}v_{h}\right)+\tilde{b}\left(u_{h}^{n-1},e_{h}^{n},I_{h}^{*}v_{h}\right)=\left(E_{n},I_{h}^{*}v_{h}\right), \end{array}$$

for all $\left(v_{h},q_{h}\right)\in \left(X_{h},M_{h}\right)$. Setting $\left(v_{h},q_{h}\right)=\left(d_{t}e_{h}^{n},\mu_{h}^{n}\right)\Delta t$ in (47), we obtain

(48) $$\begin{array}{cc} \Delta t∥d_{t}e_{h}^{n}{∥}_{0,h}^{2} & +\frac{\nu }{2}(∥{\tilde{A}}_{h}^{\frac{1}{2}}e_{h}^{n}{∥}_{0}^{2}-∥{\tilde{A}}_{h}^{\frac{1}{2}}e_{h}^{n-1}{∥}_{0}^{2})+\frac{\beta }{2}\left(C_{h}\left(\mu_{h}^{n},\mu_{h}^{n}\right)-C_{h}\left(\mu_{h}^{n-1},\mu_{h}^{n-1}\right)\right)\\ \; & +\tilde{b}\left(e_{h}^{n-1},u_{h}\left(t_{n}\right),I_{h}^{*}d_{t}e_{h}^{n}\right)\Delta t+\tilde{b}\left(u_{h}\left(t_{n-1}\right),e_{h}^{n-1},I_{h}^{*}d_{t}e_{h}^{n}\right)\Delta t\\ \; & +\tilde{b}\left(e_{h}^{n-1},e_{h}^{n},I_{h}^{*}d_{t}e_{h}^{n}\right)\Delta t=\left(E_{n},I_{h}^{*}d_{t}e_{h}^{n}\right)\Delta t \end{array}$$

From (5) and Lemma 1, we get that

$$\begin{array}{cc} |\tilde{b}\left(e_{h}^{n-1},u_{h}\left(t_{n}\right),I_{h}^{*}d_{t}e_{h}^{n}\right)| & +|\tilde{b}\left(u_{h}\left(t_{n-1}\right),e_{h}^{n-1},I_{h}^{*}d_{t}e_{h}^{n}\right)|\\ \leq \frac{1}{8}{∥d_{t}e_{h}^{n}∥}_{0,h}^{2} & +\kappa \left(∥P_{h}B\left(\cdot ,u_{h}\left(t_{n}\right)\right){∥}_{*}^{2}+{∥P_{h}B\left(u_{h}\left(t_{n-1}\right),\cdot \right)∥}_{*}^{2}\right){∥{\tilde{A}}_{h}^{\frac{1}{2}}e_{h}^{n-1}∥}_{0}^{2},\\ |b(e_{h}^{n-1},e_{h}^{n},I_{h}^{*}d_{t}e_{h}^{n})|\Delta t & \leq c∥{\tilde{A}}_{h}^{\frac{1}{2}}e_{h}^{n}{∥}_{0}∥{\tilde{A}}_{h}^{\frac{1}{2}}e_{h}^{n-1}{∥}_{0}^{2}+\kappa ∥{\tilde{A}}_{h}^{\frac{1}{2}}e_{h}^{n}{∥}_{0}^{2}{∥{\tilde{A}}_{h}^{\frac{1}{2}}e_{h}^{n-1}∥}_{0},\\ \left|(E_{n},I_{h}^{*}d_{t}e_{h}^{n})\right| & \leq \frac{1}{4}∥d_{t}e_{h}^{n}{∥}_{0,h}^{2}+c{∥E_{n}∥}_{0}^{2}. \end{array}$$

Combining (48) with the above estimates and using Lemma 7 yields

(49) $$\begin{array}{cc} ∥{\tilde{A}}_{h}^{\frac{1}{2}}e_{h}^{n}{∥}_{0}^{2}-{∥{\tilde{A}}_{h}^{\frac{1}{2}}e_{h}^{n-1}∥}_{0}^{2} & +\nu^{-1}\beta \left(C_{h}\left(\mu_{h}^{n},\mu_{h}^{n}\right)-C_{h}\left(\mu_{h}^{n-1},\mu_{h}^{n-1}\right)\right)+\nu^{-1}{∥d_{t}e_{h}^{n}∥}_{0,h}^{2}\Delta t\\ \; & \leq \kappa ((∥P_{h}B\left(\cdot ,u_{h}\left(t_{n}\right)\right){∥}_{*}^{2}+∥P_{h}B\left(u_{h}\left(t_{n-1}\right),\cdot \right){∥}_{*}^{2})\\ \; & +∥{\tilde{A}}_{h}^{\frac{1}{2}}e_{h}^{n}{∥}_{0}∥{\tilde{A}}_{h}^{\frac{1}{2}}e_{h}^{n-1}{∥}_{0}^{2}+c∥{\tilde{A}}_{h}^{\frac{1}{2}}e_{h}^{n}{∥}_{0}^{2}∥{\tilde{A}}_{h}^{\frac{1}{2}}e_{h}^{n-1}{∥}_{0}+c{∥E_{n}∥}_{0,h}^{2})\Delta t. \end{array}$$

Summing (48) from n=1 to n=m and applying Lemmas 5 and 7, we obtain

$$\begin{array}{cc} ∥{\tilde{A}}_{h}^{\frac{1}{2}}e^{m}{∥}_{0}^{2} & +\nu^{-1}\Delta t\sum_{n=1}^{m}(∥d_{t}e_{h}^{n}{∥}_{0,h}^{2}\\ \; & \leq \kappa \sum_{n=1}^{m}(\Delta t(∥P_{h}B\left(\cdot ,u_{h}\left(t_{n}\right)\right){∥}_{*}^{2}+∥P_{h}B\left(u_{h}\left(t_{n-1}\right),\cdot \right){∥}_{*}^{2})∥{\tilde{A}}_{h}^{\frac{1}{2}}e_{h}^{n-1}{∥}_{0}^{2}\\ \; & \left(∥{\tilde{A}}_{h}^{\frac{1}{2}}e_{h}^{n}{∥}_{0}∥{\tilde{A}}_{h}^{\frac{1}{2}}e_{h}^{n-1}{∥}_{0}^{2}+∥{\tilde{A}}_{h}^{\frac{1}{2}}e_{h}^{n}{∥}_{0}^{2}{∥{\tilde{A}}_{h}^{\frac{1}{2}}e_{h}^{n-1}∥}_{0}\right)+\Delta t{∥E_{n}∥}_{0,h}^{2})\leq \kappa . \end{array}$$

Combining the above estimate with (14) which yields(46).  □

**Lemma** **9.** *Under the assumptions of Lemma 7, we have that*

(50) $$\tau \left(t_{m}\right){∥e_{h}^{m}∥}_{0,h}^{2}+\Delta t\sum_{n=1}^{m}\left(∥e_{h}^{n}{∥}_{0,h}^{2}+\tau \left(t_{n}\right){∥{\tilde{A}}_{h}^{\frac{1}{2}}e_{h}^{n}∥}_{0}^{2}+\tau \left(t_{n}\right)C_{h}\left(\mu_{h}^{n},\mu_{h}^{n}\right)\right)\leq \kappa \Delta t^{2}.$$

*for all 1≤m≤N.*

**Proof.**  Setting $v=e_{h}^{n},\;\;q=\mu_{h}^{n},\;\;z^{n}=e_{h}^{n}$ in (38) and $v_{h}=\Phi_{h}^{n-1},\;\;q_{h}=\Psi_{h}^{n-1}$ in (28) we obtain

(51) $$\begin{array}{cc} \left(e_{h}^{n},I_{h}^{*}d_{t}\Phi_{h}^{n}\right) & -{\tilde{B}}_{h}\left(\left(e_{h}^{n},\mu_{h}^{n}\right);\left(I_{h}^{*}\Phi_{h}^{n-1},\Psi_{h}^{n-1}\right)\right)-\tilde{b}\left(e_{h}^{n},u_{h}\left(t_{n}\right),I_{h}^{*}\Phi_{h}^{n-1}\right)\\ \; & -\tilde{b}\left(u_{h}\left(t_{n-1}\right),e_{h}^{n},I_{h}^{*}\Phi_{h}^{n-1}\right)=\left(e_{h}^{n},I_{h}^{*}e_{h}^{n}\right), \end{array}$$

(52) $$\begin{array}{cc} \left(d_{t}e^{n},I_{h}^{*}\Phi_{h}^{n-1}\right) & +{\tilde{B}}_{h}\left(\left(e_{h}^{n},\mu_{h}^{n}\right);\left(I_{h}^{*}\Phi_{h}^{n-1},\Psi_{h}^{n-1}\right)\right)+\tilde{b}\left(u_{h}^{n-1},e_{h}^{n},I_{h}^{*}\Phi_{h}^{n-1}\right)\\ \; & +\tilde{b}\left(e_{h}^{n-1},u_{h}\left(t_{n}\right),I_{h}^{*}\Phi^{n-1}\right)=\left(E_{n},I_{h}^{*}\Phi_{h}^{n-1}\right). \end{array}$$

Adding (52) to (51), we obtain

(53) $$\begin{array}{cc} ∥e_{h}^{n}{∥}_{0,h}^{2} & =\frac{1}{\Delta t}\left(\left(e_{h}^{n},I_{h}^{*}\Phi^{n}\right)-\left(e_{h}^{n-1},I_{h}^{*}\Phi^{n-1}\right)\right)-\tilde{b}\left(d_{t}e_{h}^{n},u_{h}\left(t_{n}\right),I_{h}^{*}\Phi^{n-1}\right)\Delta t\\ \; & -\tilde{b}\left(e^{n-1},e^{n},I_{h}^{*}\Phi_{h}^{n-1}\right)-\left(E_{n},I_{h}^{*}\Phi_{h}^{n-1}\right). \end{array}$$

It follows from (14) and (15) that

$$\begin{array}{cc} |\tilde{b}\left(e_{h}^{n-1},e_{h}^{n},I_{h}^{*}\Phi_{h}^{n-1}\right)| & \leq c∥{\tilde{A}}_{h}^{\frac{1}{2}}e_{h}^{n-1}{∥}_{0}∥{\tilde{A}}_{h}^{\frac{1}{2}}e_{h}^{n}{∥}_{0}{∥{\tilde{A}}_{h}^{\frac{1}{2}}\Phi_{h}^{n-1}∥}_{0},\\ |\tilde{b}\left(d_{t}e_{h}^{n},u_{h}\left(t_{n}\right),I_{h}^{*}\Phi_{h}^{n-1}\right)| & \leq c∥d_{t}e_{h}^{n}{∥}_{0,h}∥P_{h}\left(\cdot ,u_{h}\left(t_{n}\right)\right){∥}_{*}{∥{\tilde{A}}_{h}^{\frac{1}{2}}\Phi_{h}^{n-1}∥}_{0},\\ ∥\left(E_{n},I_{h}^{*}\Phi_{h}^{n-1}\right){∥}_{0} & \leq ∥{\tilde{A}}_{h}^{-1}E_{n}{∥}_{0}{∥{\tilde{A}}_{h}\Phi_{h}^{n-1}∥}_{0}. \end{array}$$

Combining (53) with the above estimates yields

(54) $$\begin{array}{cc} ∥e_{h}^{n}{∥}_{0,h}^{2}\Delta t & =\left(e_{h}^{n},I_{h}^{*}\Phi_{h}^{n}\right)-\left(e_{h}^{n-1},I_{h}^{*}\Phi_{h}^{n-1}\right)+c∥{\tilde{A}}_{h}^{\frac{1}{2}}e_{h}^{n-1}{∥}_{0}∥{\tilde{A}}_{h}^{\frac{1}{2}}e_{h}^{n}{∥}_{0}{∥{\tilde{A}}_{h}^{\frac{1}{2}}\Phi^{n-1}∥}_{0}\Delta t\\ \; & +c\Delta t^{2}∥d_{t}e_{h}^{n}{∥}_{0,h}∥P_{h}B\left(\cdot ,u_{h}\left(t_{n}\right)\right){∥}_{*}∥{\tilde{A}}_{h}^{\frac{1}{2}}\Phi_{h}^{n-1}{∥}_{0}+∥{\tilde{A}}_{h}^{-1}E_{n}{∥}_{0}{∥{\tilde{A}}_{h}\Phi_{h}^{n-1}∥}_{0}\Delta t, \end{array}$$

with $e_{h}^{0}=\Phi^{m}=0$. Summing (54) from n=1 to n=m and applying Lemma 6, we obtain

(55) $$\begin{array}{cc} \Delta t\sum_{n=1}^{m}{∥e_{h}^{n}∥}_{0,h}^{2} & \leq \kappa \Delta t\left(\sum_{n=1}^{m}∥{\tilde{A}}_{h}^{\frac{1}{2}}e_{h}^{n}{∥}_{0}^{2}+\sum_{n=1}^{m}{∥{\tilde{A}}_{h}^{-1}E_{n}∥}_{0}^{2}\right)\\ \; & +c\Delta t^{2}\left(\sum_{n=1}^{m}{∥d_{t}e_{h}^{n}∥}_{0,h}^{2}\Delta t\right)\left(\sum_{n=1}^{m}{∥P_{h}B\left(\cdot ,u_{h}\left(t_{n}\right)\right)∥}_{*}^{2}\Delta t\right). \end{array}$$

Applying Lemmas 4, 5, 7 and 8 in (55), we get and

(56) $$\Delta t\sum_{n=1}^{m}{∥e_{h}^{n}∥}_{0,h}^{2}\leq \kappa \Delta t^{2},\;\;\forall 1\leq m\leq N.$$

Now, multiplying (55) by $\tau (t_{n})$ and noting

$$\tau \left(t_{n}\right)\leq \Delta t+\tau \left(t_{n-1}\right),\;\;\Delta t\leq \tau \left(t_{n-1}\right),\;\;2\leq n\leq N,\;\;e_{h}^{n-1}=0,\;\;forn=1,$$

we get

(57) $$\begin{array}{cc} \tau \left(t_{n}\right){∥e_{h}^{n}∥}_{0}^{2} & -\tau \left(t_{n-1}\right)∥e_{h}^{n-1}{∥}_{0}^{2}+\tau \left(t_{n}\right)(\nu ∥{\tilde{A}}_{h}^{\frac{1}{2}}e_{h}^{n}{∥}_{0}^{2}+\beta C_{h}\left(\mu_{h}^{n},\mu_{h}^{n}\right))\Delta t\\ \; & \leq c∥P_{h}B\left(\cdot ,u_{h}\left(t_{n}\right)\right){∥}_{*}^{2}\tau \left(t_{n-1}\right){∥e_{h}^{n-1}∥}_{0}^{2}\Delta t\\ \; & +c\left(∥e_{h}^{n-1}{∥}_{0}^{2}+\tau \left(t_{n}\right){∥{\tilde{A}}_{h}^{-1/2}E_{n}∥}_{0}^{2}\right)\Delta t. \end{array}$$

Summing (57) from n=1 to n=m and applying Lemmas 3 and 5, we deduce that

(58) $$\begin{array}{cc} \tau \left(t_{m}\right){∥e_{h}^{m}∥}_{0,h}^{2} & +\Delta t\sum_{n=1}^{m}\tau \left(t_{n}\right)(\nu ∥{\tilde{A}}_{h}^{\frac{1}{2}}e_{h}^{n}{∥}_{0}^{2}+\beta C_{h}\left(\mu_{h}^{n},\mu_{h}^{n}\right))\\ \; & \leq \kappa \Delta t\sum_{n=1}^{m}\left(∥e_{h}^{n-1}{∥}_{0,h}^{2}+\tau \left(t_{n}\right){∥A_{h}^{-1/2}E_{n}∥}_{0}^{2}\right). \end{array}$$

Applying Lemma 4 and (56) in (58), we have

(59) $$\tau \left(t_{m}\right){∥e_{h}^{m}∥}_{0,h}^{2}+\Delta t\sum_{n=0}^{m}\tau \left(t_{n}\right)\left(\nu ∥{\tilde{A}}_{h}^{\frac{1}{2}}e_{h}^{n}{∥}_{0}^{2}+\beta C_{h}\left(\mu_{h}^{n},\mu_{h}^{n}\right)\right)\leq \kappa \Delta t^{2}.$$

this and (56) yield (50).  □

**Lemma** **10.** *Under the assumptions of Lemma 7, the error $\left(e_{h}^{n},\mu_{h}^{n}\right)$ satisfies the following bound:*

(60) $$\tau^{2}\left(t_{m}\right)∥{\tilde{A}}_{h}^{\frac{1}{2}}e_{h}^{m}{∥}_{0}^{2}+\Delta t\sum_{n=1}^{m}\tau^{2}\left(t_{n}\right){∥d_{t}e_{h}^{n}∥}_{0,h}^{2}\leq \kappa \Delta t^{2},\;\;1\leq m\leq N.$$

**Proof.**  Multiplying (49) by $\tau^{2}\left(t_{n}\right),$ noting $\tau^{2}\left(t_{n}\right)\leq \tau^{2}\left(t_{n-1}\right)+3\tau \left(t_{n-1}\right)\Delta t$ and using Theorem 4, we have that

(61) $$\begin{array}{cc} \tau^{2}\left(t_{n}\right)(∥{\tilde{A}}_{h}^{\frac{1}{2}}e_{h}^{n}{∥}_{0}^{2} & +\nu^{-1}\beta C_{h}\left(\mu_{h}^{n},\mu_{h}^{n}\right))-\tau^{2}\left(t_{n-1}\right)(∥{\tilde{A}}_{h}^{\frac{1}{2}}e_{h}^{n-1}{∥}_{0}^{2}+\nu^{-1}\beta C_{h}\left(\mu_{h}^{n-1},\mu_{h}^{n-1}\right))\\ \;\; & +\nu^{-1}\tau^{2}\left(t_{n}\right)∥d_{t}e_{h}^{n}{∥}_{0,h}^{2}\Delta t\leq c\tau^{2}\left(t_{n}\right){∥E_{n}∥}_{0,h}^{2}\Delta t\\ \; & +c\tau \left(t_{n-1}\right)(∥{\tilde{A}}_{h}^{\frac{1}{2}}e_{h}^{n-1}{∥}_{0}^{2}+\nu^{-1}\beta C_{h}\left(\mu_{h}^{n-1},\mu_{h}^{n-1}\right))\Delta t. \end{array}$$

Summing (61) from n=1 to n=m and applying Lemmas 4 and 9, we have

(62) $$\tau^{2}\left(t_{m}\right)∥{\tilde{A}}_{h}^{\frac{1}{2}}e_{h}^{m}{∥}_{0}^{2}+\Delta t\sum_{n=1}^{m}\tau^{2}\left(t_{n}\right){∥d_{t}e_{h}^{n}∥}_{0,h}^{2}\leq \kappa \Delta t^{2}.$$

 □

**Lemma** **11.** *Under the assumptions of Lemma 7, the error $\mu_{h}^{n}={\bar{p}}_{h}\left(t_{n}\right)-p_{h}^{n}$ satisfies the following bound:*

(63) $$\tau^{2}\left(t_{n}\right){∥{\bar{p}}_{h}\left(t_{n}\right)-p_{h}^{n}∥}_{0}^{2}\leq \kappa \Delta t,\;\;1\leq m\leq N.$$

**Proof.**  From Theorem 2, (5), (14) and (28), we deduce that

$$\begin{array}{cc} ∥\mu_{h}^{n}{∥}_{0}\leq c{∥{\tilde{A}}_{h}^{\frac{1}{2}}e_{h}^{n}∥}_{0} & +c∥d_{t}e_{h}^{n}{∥}_{0,h}+c\left(∥{\tilde{A}}_{h}^{\frac{1}{2}}e_{h}^{n-1}{∥}_{0}+{∥{\tilde{A}}_{h}^{\frac{1}{2}}u_{h}\left(t_{n-1}\right)∥}_{0}\right){∥{\tilde{A}}_{h}^{\frac{1}{2}}e_{h}^{n}∥}_{0}\\ \; & +c∥{\tilde{A}}_{h}^{\frac{1}{2}}u_{h}\left(t_{n}\right){∥}_{0}∥{\tilde{A}}_{h}^{\frac{1}{2}}e_{h}^{n-1}{∥}_{0}+c{∥E_{n}∥}_{0,h}. \end{array}$$

Hence

(64) $$\begin{array}{cc} \tau^{2}\left(t_{n}\right){∥\mu_{h}^{n}∥}_{0}^{2}\Delta t & \leq \kappa \left(\tau \left(t_{n}\right)∥{\tilde{A}}_{h}^{\frac{1}{2}}e_{h}^{n}{∥}_{0}^{2}+\tau \left(t_{n-1}\right){∥{\tilde{A}}_{h}^{\frac{1}{2}}e_{h}^{n-1}∥}_{0}^{2}\right)\Delta t+\kappa {∥{\tilde{A}}_{h}^{\frac{1}{2}}e_{h}^{n-1}∥}_{0}^{2}\Delta t^{2}\\ \;\; & +c\tau^{2}\left(t_{n}\right)∥d_{t}e_{h}^{n}{∥}_{0,h}^{2}\Delta t+c\tau^{2}\left(t_{n}\right){∥E_{n}∥}_{0,h}^{2}\Delta t. \end{array}$$

Summing (64) for *n* from n=1 to n=m and applying Lemmas 4, 9 and 10, we deduce that

(65) $$\Delta t\sum_{n=1}^{m}\tau^{2}\left(t_{n}\right){∥\mu_{h}^{n}∥}_{0}^{2}\leq \kappa \Delta t^{2},\;\;0\leq m\leq N,$$

which yields (63).  □

**Theorem** **5.** *Under the assumptions of Lemma 7, the error $\left(u\left(t_{n}\right)-u_{h}^{n},p\left(t_{n}\right)-p_{h}^{n}\right)$ satisfies the following optimal bounds:*

(66) $$\begin{array}{c} \Delta t\sum_{n=1}^{N}∥{\tilde{A}}_{h}^{\frac{1}{2}}\left(u\left(t_{n}\right)-u_{h}^{n}\right){∥}_{0}^{2}+\tau \left(t_{m}\right){∥{\tilde{A}}_{h}^{\frac{1}{2}}\left(u\left(t_{m}\right)-u_{h}^{m}\right)∥}_{0}^{2}\leq \kappa \left(h^{2}+\Delta t\right),\\ \Delta t\sum_{n=1}^{N}\tau \left(t_{n}\right)∥p\left(t_{n}\right)-p_{h}^{n}{∥}_{0}^{2}+\tau^{2}\left(t_{m}\right){∥p\left(t_{m}\right)-p_{h}^{m}∥}_{0}^{2}\leq \kappa \left(h^{2}+\Delta t\right), \end{array}$$

*for all $t_{m}\in \left(0,T\right]$.*

**Proof.**  Integration by parts directly can show

(67) $$\begin{array}{cc} \Delta t∥{\tilde{A}}_{h}^{\frac{1}{2}}\left(u\left(t_{n}\right)-u_{h}\left(t_{n}\right)\right){∥}_{0}^{2} & \leq 2\int_{t_{n-1}}^{t_{n}}{∥{\tilde{A}}_{h}^{\frac{1}{2}}\left(u-u_{h}\right)∥}_{0}^{2}dt\\ \; & +\int_{t_{n-1}}^{t_{n}}{\left(t-t_{n-1}\right)}^{2}{∥{\tilde{A}}_{h}^{\frac{1}{2}}\left(u_{t}-u_{ht}\right)∥}_{0}^{2}dt. \end{array}$$

Summing (67) from n=1 to n=N, using (19) and noting $t-t_{n-1}\leq \tau \left(t\right)$, we obtain

(68) $$\Delta t\sum_{n=1}^{N}∥{\tilde{A}}_{h}^{\frac{1}{2}}\left(u\left(t_{n}\right)-u_{h}\left(t_{n}\right)\right){∥}_{0}^{2}\leq 2\int_{0}^{T}{∥{\tilde{A}}_{h}^{\frac{1}{2}}\left(u-u_{h}\right)∥}_{0}^{2}dt+\kappa h^{2},$$

Combining (68) with (14), Theorem 3, Lemmas 7 and 9 yields the first estimate in (66).

(69) $$\begin{array}{c} \Delta t\sum_{n=1}^{N}∥{\tilde{A}}_{h}^{\frac{1}{2}}\left(u\left(t_{n}\right)-u_{h}^{n}\right){∥}_{0}^{2}+\tau \left(t_{m}\right){∥{\tilde{A}}_{h}^{\frac{1}{2}}\left(u\left(t_{m}\right)-u_{h}^{m}\right)∥}_{0}^{2}\leq \kappa \left(h^{2}+\Delta t\right), \end{array}$$

Moreover, by (5), (9), (11), (14) and (20), we deduce that

(70) $$\int_{0}^{t}{∥p_{h}∥}_{0}^{2}ds\leq c\int_{0}^{t}\left(∥u_{ht}{∥}_{0}^{2}+∥{\tilde{A}}_{h}^{\frac{1}{2}}u_{h}{∥}_{0}^{4}+{∥f∥}_{0}^{2}\right)ds\leq \kappa ,\;\;0\leq t\leq T.$$

Hence, we obtain from Theorems 1 and 3 that

(71) $$\begin{array}{cc} \tau \left(t_{1}\right)∥p\left(t_{1}\right) & -{\bar{p}}_{h}\left(t_{1}\right){∥}_{0}\leq \Delta t^{1/2}\left(\tau^{1/2}\left(t_{1}\right){∥p\left(t_{1}\right)∥}_{0}+{\left(\int_{0}^{t_{1}}{∥p_{h}∥}_{0}^{2}ds\right)}^{1/2}\right)\\ \; & \leq \kappa \Delta t^{1/2}, \end{array}$$

(72) $$\begin{array}{cc} \tau \left(t_{n}\right)∥p\left(t_{n}\right) & -{\bar{p}}_{h}\left(t_{n}\right){∥}_{0}\leq \tau \left(t_{n}\right)∥p\left(t_{n}\right)-\bar{p}\left(t_{n}\right){∥}_{0}+\tau \left(t_{n}\right){∥\bar{p}\left(t_{n}\right)-{\bar{p}}_{h}\left(t_{n}\right)∥}_{0}\\ \; & \leq \frac{2}{\Delta t}\int_{t_{n-1}}^{t_{n}}\left(t-t_{n-1}\right)\tau \left(t\right)∥p_{t}{∥}_{0}dt+\frac{2}{\Delta t}\int_{t_{n-1}}^{t_{n}}\tau \left(t\right){∥p-p_{h}∥}_{0}dt\\ \; & \leq c\Delta t^{1/2}{\left(\int_{t_{n-1}}^{t_{n}}\tau^{2}\left(t\right){∥p_{t}∥}_{0}^{2}dt\right)}^{1/2}+\kappa h\leq \kappa \left(h+\Delta t^{1/2}\right). \end{array}$$

Combining (63) with (65)–(72) yields

(73) $$\tau^{2}\left(t_{m}\right){∥p\left(t_{m}\right)-p_{h}^{m}∥}_{0}^{2}\leq \kappa \left(h^{2}+\Delta t\right),$$

for all $t_{m}\in \left(0,T\right]$.

Besides, noting that

(74) $$\begin{array}{cc} \Delta t\sum_{n=2}^{N}\tau \left(t_{n}\right){∥p\left(t_{n}\right)-{\bar{p}}_{h}\left(t_{n}\right)∥}_{0}^{2} & \leq \sum_{n=2}^{N}\frac{2\tau (t_{n})}{\Delta t}({∥\int_{t_{n-1}}^{t_{n}}\left(p\left(t_{n}\right)-p\left(t\right)\right)dt∥}_{0}^{2}\\ +∥\int_{t_{n-1}}^{t_{n}}\left(p-p_{h}\right){dt∥}_{0}^{2}) & \leq 4\Delta t\int_{t_{1}}^{T}\tau^{2}\left(t\right)∥p_{t}{∥}_{0}^{2}dt+4\int_{t_{1}}^{T}\tau \left(t\right){∥p-p_{h}∥}_{0}^{2}dt. \end{array}$$

Recalling (Lemma 4.3) in [12], we have

(75) $$\begin{array}{cc} \left(u_{t}-u_{ht},I_{h}^{*}v\right) & +{\tilde{B}}_{h}\left(\left(e_{h},\mu_{h}\right);\left(I_{h}^{*}v,q\right)\right)+\tilde{b}\left(u,u-u_{h},I_{h}^{*}v\right)+\tilde{b}\left(u-u_{h},u,I_{h}^{*}v\right)\\ \; & -\tilde{b}\left(u-u_{h},u-u_{h},I_{h}^{*}v\right)=0\;\;\forall \left(v,q\right)\in \left(X_{h},M_{h}\right), \end{array}$$

where $e_{h}={\tilde{R}}_{h}\left(u,p\right)-u_{h},\mu_{h}={\tilde{Q}}_{h}\left(u,p\right)-p_{h}.$ Using (9), (11), (14) and (75), we obtain

$$\int_{0}^{T}\tau \left(t\right){∥\mu_{h}∥}_{0}^{2}dt\leq c\int_{0}^{T}\left(\tau \left(t\right)∥u_{t}-u_{ht}{∥}_{0}^{2}+∥{\tilde{A}}_{h}^{\frac{1}{2}}\left(u-u_{h}\right){∥}_{0}^{2}{(∥u∥}^{2}+∥{\tilde{A}}_{h}^{\frac{1}{2}}u_{h}{∥}_{0}^{2})\right)dt.$$

It follows from (5), (7), (18)–(20), and the above estimate that

(76) $$\begin{array}{cc} \int_{0}^{T}\tau \left(t\right){∥p-p_{h}∥}_{0}^{2}dt & \leq 2\int_{0}^{T}\tau \left(t\right)(∥p-{\tilde{Q}}_{h}{\left(u,p\right)∥}_{0}^{2}+∥\mu_{h}{∥}_{0}^{2})dt\\ \; & \leq ch^{2}\int_{0}^{T}{(∥Au∥}_{0}^{2}+{∥p∥}_{1}^{2})dt+\kappa h^{2}\leq \kappa h^{2}. \end{array}$$

Substituting (76) into (74) and using (73) and Theorem 1, we get

(77) $$\Delta t\sum_{n=1}^{N}\tau \left(t_{n}\right){∥p\left(t_{n}\right)-{\bar{p}}_{h}\left(t_{n}\right)∥}_{0}^{2}\leq \kappa \left(h^{2}+\Delta t\right).$$

Combining (77) with (65), we have

(78) $$\Delta t\sum_{n=1}^{N}\tau \left(t_{n}\right){∥p\left(t_{n}\right)-p_{h}^{n}∥}_{0}^{2}\leq \kappa \left(h^{2}+\Delta t\right).$$

This and (69) yield (66).  □

## 6. Single Numerical Example

In this section, some numerical results are computed to test the rationality of the theoretical analysis. Because it is difficult to obtain the analytical solution of the general problem governed by the Navier-Stokes equation, we show the relevant numerical results through an example with analytical solution for simplicity. So we consider the following model problem in the unit square area [0,1]×[0,1]. Here this example might as well takes ν as 0.01. Only the velocities and pressure are given here. The right term *f* of the equations can be obtained by bringing the relationship between p(x) and $u\left(x,y\right)=(u_{1}\left(x,y\right),u_{2}\left(x,y\right))$ into the NS equations, and the initial values of $u_{1}\left(x,y\right),u_{2}\left(x,y\right)$ and p(x,y) can be obtained by bringing t=0 into the calculation.

Now, consider a unit square domain with an exact solution given by

(79) $$\begin{array}{cc} \; & u\left(x,y\right)=(u_{1}\left(x,y\right),u_{2}\left(x,y\right)),\\ \; & u_{1}\left(x,y\right)=e^{-8\pi^{2}\nu }10x^{2}{\left(x-1\right)}^{2}y\left(y-1\right)\left(2y-1\right),\\ \; & u_{2}\left(x,y\right)=-e^{-8\pi^{2}\nu }10x\left(x-1\right)\left(2x-1\right)y^{2}{\left(y-1\right)}^{2},\\ \; & p\left(x,y\right)=e^{-16\pi^{2}\nu }10\left(2x-1\right)\left(2y-1\right). \end{array}$$

*f* is determined by (3). It can be verified that such $u_{1}\left(x,y\right),u_{2}\left(x,y\right)$ satisfy the non divergence condition.

For simplicity, we can record the time and spatial discretization of the problem as follows:

(80) $$\left\{\begin{array}{c} Au^{n+1}+N\left(u^{n}\right)u^{n+1}+Bp^{n+1}=f^{n+1},\\ -Bu^{n+1}+\beta C=0. \end{array}\right.$$

where the matrices in (80) correspond to the differential operators: $A∽-\mathrm{diag}\left(v\Delta +1/\Delta t\right),N\left(u^{n}\right)∼u^{n}\cdot \nabla ,B∼\nabla ,C∼J_{h}\mathrm{div},C∼C_{h}\left(\cdot ,\cdot \right)$, and *I* is the identity matrix. The right-hand side $f^{n+1}$ contains the source term.

To make the next iterations less complex, here are a few new notations. Let $W=u^{n+1}$, $q=p^{n+1}$, then we can further record the above equation as follows:

$$vA+N\left(w\right)w+Bq=f-B^{T}w+\beta C=0.$$

Besides, in order to improve the calculation efficiency, we can generally adopt Newton iterative method to solve the above nonlinear problems. The typical calculation steps are as follows:

(81) $$\begin{array}{cc} \; & \left(1\right)R=f-\left(vAv^{\mathrm{old}\;}+N\left(v^{\mathrm{old}\;}\right)\right)v^{\mathrm{old}\;}-Bq^{\mathrm{old}\;,}\;r=-B^{T}v^{\mathrm{old}\;};\\ \; & \left(2\right)\left(vAv^{\mathrm{mid}\;}+N\left(v^{\mathrm{old}\;}\right)\right)v^{\mathrm{mid}\;}+N\left(v^{\mathrm{mid}\;}\right)v^{\mathrm{old}\;}+Bq^{\mathrm{mid}\;}=R,\;B^{T}v^{\mathrm{mid}\;}=r;\\ \; & \left(3\right)v^{\mathrm{new}\;}=v^{\mathrm{old}\;}+v^{\mathrm{mid}\;},\;q^{\mathrm{new}\;}=q^{\mathrm{old}\;}+q^{\mathrm{mid}\;}. \end{array}$$

It is worth noting that since Newton iterative method requires high initial values, we need to use the following Picard method to obtain the initial values of Newton iterative method:

(82) $$\begin{array}{cc} \; & \left(1\right)R=f-\left(vAv^{\mathrm{old}\;}+N\left(v^{\mathrm{old}\;}\right)\right)v^{\mathrm{old}\;}-Bq^{\mathrm{old}\;},\;r=-B^{T}v^{\mathrm{old}\;};\\ \; & \left(2\right)\left(vAv^{\mathrm{mid}\;}+N\left(v^{\mathrm{old}\;}\right)\right)v^{\mathrm{mid}\;}+Bq^{\mathrm{mid}\;}=R,\;B^{T}v^{\mathrm{mid}\;}=r;\\ \; & \left(3\right)v^{\mathrm{new}\;}=v^{\mathrm{old}\;}+v^{\mathrm{mid}\;},\;q^{\mathrm{new}\;}=q^{\mathrm{old}\;}+q^{\mathrm{mid}\;}. \end{array}$$

In this way, we can finally transform the time-dependent Navier Stokes problem into a large-scale linear system of equations and solve it through such links: Euler time discretization → finite volume space discretization → Newton iterative transformation → Picard format transformation → large linear equations → solving equations.

**Figure 3:**
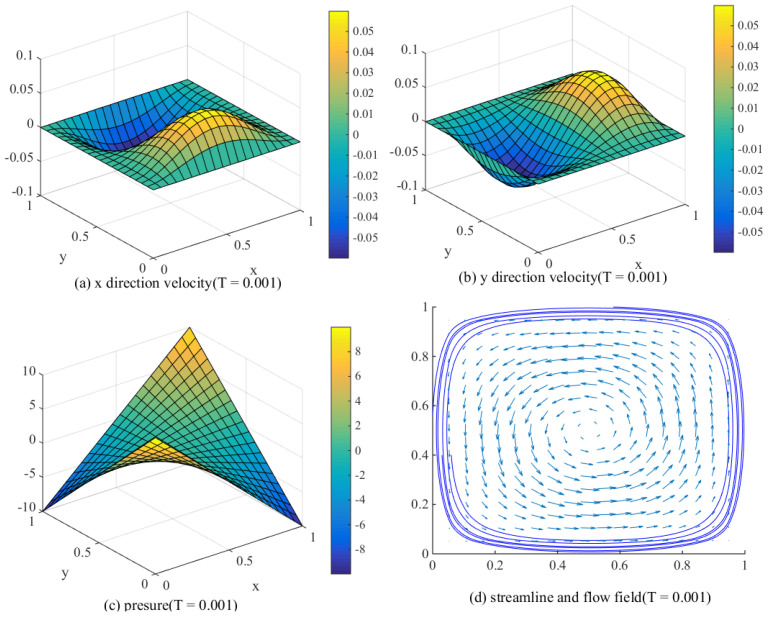
Preliminary calculated velocity and pressure.

Based on the above description, we can get some simulation results (β=6). The following Figure 3 is the result of one iteration based on the initial value. The time step here is Δt=0.001, and the spatial grid is divided into two congruent triangles on the basis of 100×100 equidistant rectangular grid. We only pay attention to that only 1/5 of the data in both *X* and *Y* directions are selected.

**Figure 4:**
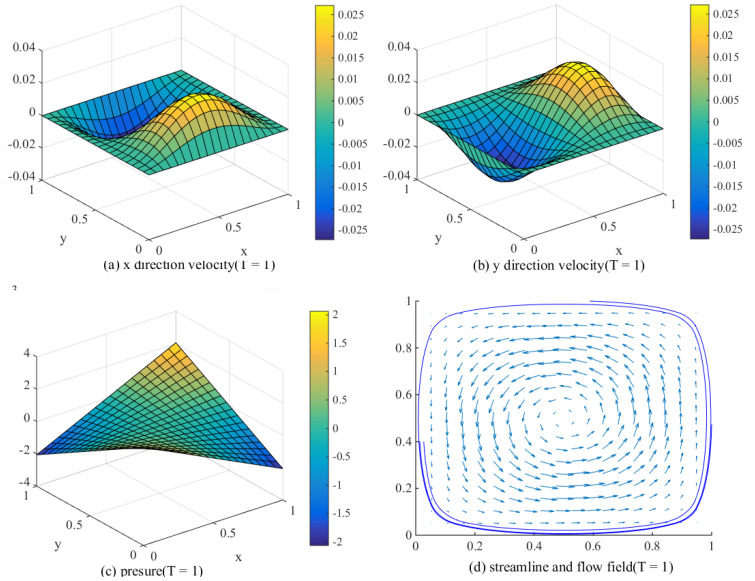
The calculated velocity and pressure at T=1.

The following Figure 4 is the result after 1000 iterations with time step Δt=0.001. Here, the reference value of streamline is the same as that when t=0.001.

Compared Figure 2 with Figure 3, it can be seen from Figure 3 that the streamline and flow field are weakened accordingly. It is easy to understand that since the interpretation constructed here is decaying, both velocity and pressure show a decaying trend. This also shows that our numerical method maintains strong robustness, and the calculation results are more intuitive.

The following table shows the $L^{2}$ error order of velocity and pressure after 1000 iterations with 20×20,40×40,80×80 grids selected under the condition of Δt=0.001.

From the above Table 1, we can see the optimal $L^{2}$ convergence rate, almost 2 for velocities and 1 for pressure are really obtained, which confirm the numerical analysis above.

**Figure 5:**
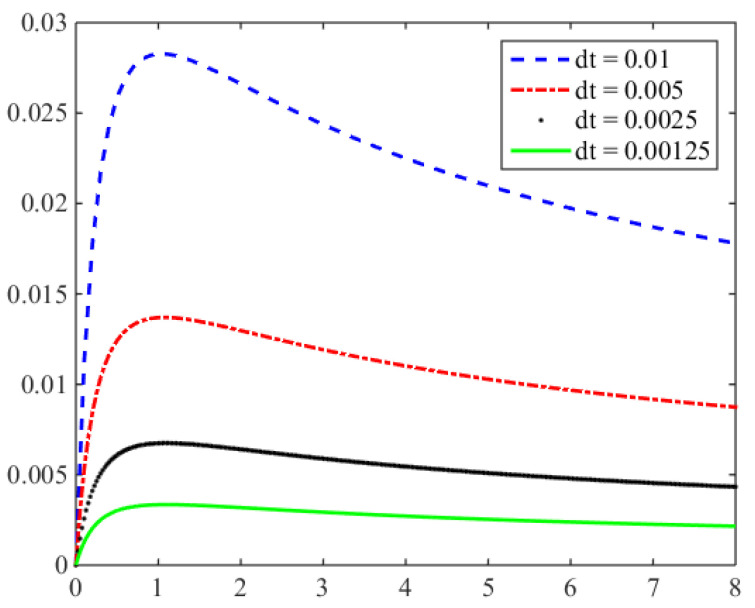
Velocity error of different time steps.

The following Figure 5 shows the error curve calculated based on 100×100 spatial grid with different time steps: Δt=0.01,0.005,0.0025,0.00125 (`dt’ in Figure 5 is Δt). It can be seen from here that the initial error tends to increase, but the error also decreases with the decrease of flow field energy, which shows that the time iteration is stable.

**Figure 6:**
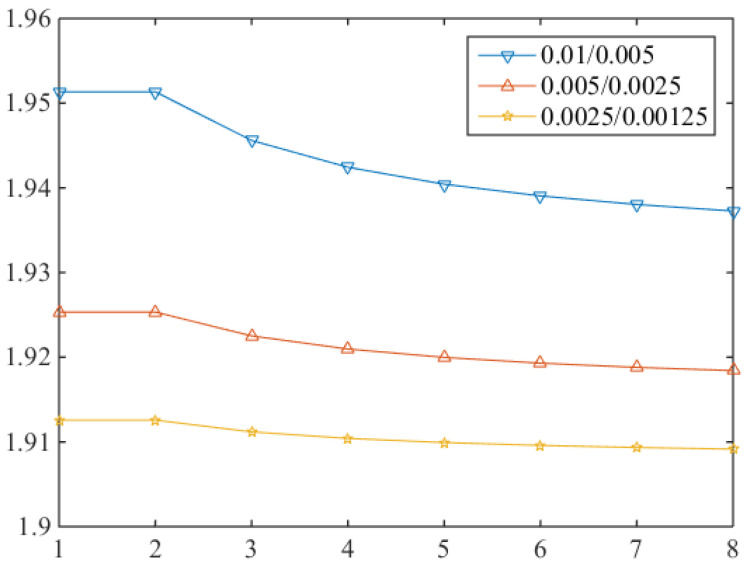
1 order convergence ratio in time direction.

The following Table 2 shows the error ratio of different time steps: dt = 0.01, 0.005, 0.0025, 0.00125:

The error ratio curve in the Figure 6 below shows the convergence of one section of time and is relatively stable.

Table 2 and Figure 6 tell us the optimal convergence rate of time is 1 which is consistent with the theoretical analysis.

Due to time constraints, our numerical results only show these. It is also worth noting that if the solution does not decay but increases, and if the growth rate is fast, the error of numerical results will increase with the increase of numerical calculation time. At that time, the method may not converge or inefficient. This requires a little attention in specific applications.

## 7. Conclusions

After detailed theoretical analysis, this article finally proves that if we use the finite volume method based on $P_{1}-P_{0}$ element to approximate the non-stationary Navier-Stokes equation, we can achieve the follow optimal numerical error estimation:

(83) $$\begin{array}{c} \Delta t\sum_{n=1}^{N}∥{\tilde{A}}_{h}^{\frac{1}{2}}\left(u\left(t_{n}\right)-u_{h}^{n}\right){∥}_{0}^{2}+\tau \left(t_{m}\right){∥{\tilde{A}}_{h}^{\frac{1}{2}}\left(u\left(t_{m}\right)-u_{h}^{m}\right)∥}_{0}^{2}\leq \kappa \left(h^{2}+\Delta t\right),\\ \;\;\Delta t\sum_{n=1}^{N}\tau \left(t_{n}\right)∥p\left(t_{n}\right)-p_{h}^{n}{∥}_{0}^{2}+\tau^{2}\left(t_{m}\right){∥p\left(t_{m}\right)-p_{h}^{m}∥}_{0}^{2}\leq \kappa \left(h^{2}+\Delta t\right). \end{array}$$

The optimal error estimate (83) shows that the time discretization of Euler method is 1 order and the space discretization is 2 order in this space-time full discretization finite volume method, which is consistent with the theoretical optimal order error estimation of $P_{1}-P_{0}$ element in solving differential equations. Although the proof process is challenging and cumbersome, the optimal result is also obvious and certain. However, this work is still helpful to reveal some special aspects of the finite volume method which is different from finite element and other methods in solving complex differential equations. Therefore, it is helpful to improve the corresponding numerical analysis theory.

In addition, with the continuous research and exploration of using neural network to solve differential equations recently [25,26,27], although very gratifying numerical results have been obtained, but the effectiveness and convergence of neural network-based methods for solving differential equations are still unclear, and there is a lack of theory. However, we know gradually that the key point of neural network in the calculation of differential equations is to use low-order functions for numerical approximation, which is similar to the basic principle of using low-order continuous functions to discretize and approximate differential equations, except that the former is global approximation while the latter is piecewise approximation. Therefore, improving and enriching the low-order function approximation theory also provides some important reference in further understanding the neural network in solving differential equations efficiently, It is worth studying.

## Figures and Tables

**Table 1:** Convergence order of spatial solution the FVM (ν=0.01, β=6).

*h*	$\frac{∥{\tilde{A}}_{h}^{\frac{1}{2}}\left(u-u_{h}\right){∥}_{0}}{∥{\tilde{A}}_{h}^{\frac{1}{2}}{u∥}_{0}}$	$\frac{∥u-u_{h}{∥}_{0}}{{∥u∥}_{0}}$	$\frac{∥p-p_{h}{∥}_{0}}{{∥p∥}_{0}}$
1/20	0.0699111	0.0035712	0.0771281
1/40	0.0394281	0.0009511	0.0440669
$\frac{1/20}{1/40}$	1.773	3.754	1.750
1/80	0.0209534	0.0002638	0.0241342
$\frac{1/40}{1/80}$	1.718	3.605	1.673

**Table 2:** Numerical results of the FVM (ν=0.005, β=10).

*t*	1	2	3	4	5	6	7	8
$\frac{\Delta t=0.01}{\Delta t=0.005}$	1.9513	1.9513	1.9456	1.9424	1.9404	1.9390	1.9380	1.9373
$\frac{\Delta t=0.05}{\Delta t=0.0025}$	1.9253	1.9253	1.9225	1.9210	1.9200	1.9193	1.9188	1.9184
$\frac{\Delta t=0.0025}{\Delta t=0.00125}$	1.9126	1.9126	1.9112	1.9104	1.9099	1.9096	1.9093	1.9092
